# Joint experimental and theoretical study of PbGa_2_S_4_ under compression†

**DOI:** 10.1039/d3tc02288a

**Published:** 2023-07-28

**Authors:** Tania Garcia-Sanchez, Samuel Gallego-Parra, Akun Liang, José Luis Rodrigo-Ramon, Alfonso Muñoz, Plácida Rodriguez-Hernandez, Javier Gonzalez-Platas, Juán Ángel Sans, Vanesa Paula Cuenca-Gotor, Hussien H. Osman, Catalin Popescu, Veaceslav Ursaki, Ion M. Tiginyanu, Daniel Errandonea, Francisco Javier Manjón

**Affiliations:** a Departamento de Ingeniería Eléctica, MALTA Consolider Team, Universitat Politècnica de València Camino de Vera, s/n. Valencia Spain tagarsan@die.upv.es +34 96 387 70 00; b Departamento de Física, MALTA Consolider Team, Universitat Politècnica de València Camino de Vera, s/n. Valencia Spain; c Departamento de Física Aplicada-ICMUV, MALTA Consolider Team, Universitat de Valencia Dr Moliner 50, Burjassot Valencia 46100 Spain; d Departamento de Física, Instituto de Materiales y Nanotecnología, MALTA Consolider Team, Universidad de La Laguna La Laguna Tenerife 38205 Spain; e ALBA-CELLS, MALTA Consolider Team, Cerdanyola del Valles (Barcelona) Cataluña 08290 Spain; f National Center for Materials Study and Testing, Technical University of Moldova Chisinau MD-2004 Republic of Moldova

## Abstract

The effect of pressure on the structural, vibrational, and optical properties of lead thiogallate, PbGa_2_S_4_, crystallizing under room conditions in the orthorhombic EuGa_2_S_4_-type structure (space group *Fddd*), is investigated. The results from X-ray diffraction, Raman scattering, and optical-absorption measurements at a high pressure beyond 20 GPa are reported and compared not only to *ab initio* calculations, but also to the related compounds α′-Ga_2_S_3_, CdGa_2_S_4_, and HgGa_2_S_4_. Evidence of a partially reversible pressure-induced decomposition of PbGa_2_S_4_ into a mixture of Pb_6_Ga_10_S_21_ and Ga_2_S_3_ above 15 GPa is reported. Thus, our measurements and calculations show a route for the high-pressure synthesis of Pb_6_Ga_10_S_21_, which is isostructural to the stable Pb_6_In_10_S_21_ compound at room pressure.

## Abstract 2

The effect of pressure on the structural, vibrational, and optical properties of lead thiogallate, PbGa_2_S_4_, crystallizing under room conditions in the orthorhombic EuGa_2_S_4_-type structure (space group *Fddd*), is investigated.

## Graphical abstract

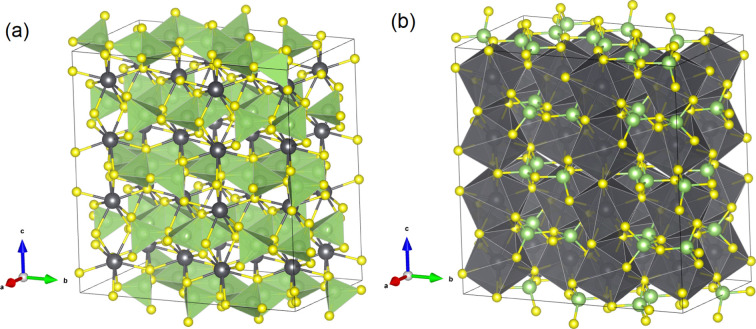

## 1 Introduction

Ternary metal chalcogenides of the A^II^B^III^_2_X^VI^_4_ family (X = S, Se, Te) can be basically divided into three subfamilies. The first one is constituted by compounds with both A and B cations showing a fourfold coordination. These compounds usually crystallize in the defect chalcopyrite, defect stannite (or defect famatinite), pseudo-cubic, and related structures that are derived from the zinc blende or wurtzite structures. Examples of those compounds are (Zn,Cd,Hg)(Al,Ga)_2_(S,Se)_4_ compounds. The second subfamily is constituted by compounds in which there is a mixture of cations with fourfold and sixfold coordination. These compounds crystallize mainly in the spinel (MgAl_2_O_4_) or in related structures, such as (Mg,Zn,Cd,Mn)In_2_(S,Se)_4_, and show similar structural characteristics to many oxospinels. The third subfamily, and the less studied one, is composed of A cations featuring a coordination much larger than six. Examples of these compounds are those crystallizing in the orthorhombic EuGa_2_S_4_-type and related structures, such as (Ca,Sr,Pb,Eu,Sm,Yb)(Al,Ga,In)_2_ (S,Se,Te)_4_.^1^

The studies in the last subfamily of EuGa_2_S_4_-type compounds have come from the interest in the development first of phosphors,^2^ and later of mid-infrared (35 μm) solid-state lasers,^3–5^ due to the large band gap, low-phonon energy, and chemical and thermal stability of these ternary sulphides. In particular, mid-infrared (mid-IR) laser radiation in PbGa_2_S_4_ has been consistently reported.^6–10^ This fact has resulted in recent studies to improve the crystal quality of this mid-IR laser material.^11,12^

**Fig. 1:**
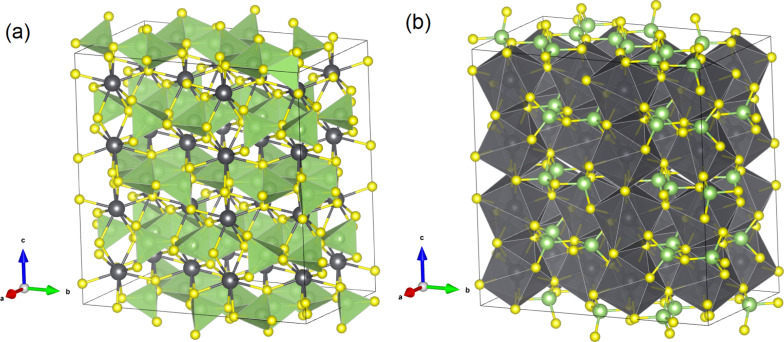
Schematic representation of the crystal structure of PbGa_2_S_4_. (a) Crystal structure highlighting GaS_4_ tetrahedral units. (b) Crystal structure highlighting square antiprismatic PbS_8_ polyhedra. PB, Ga, and S atoms are shown in black, green, and yellow colors. A figure highlighting the layered characteristic of the crystal structure is given in Fig. S2 in the ESI.†

Several works have reported the structural, vibrational, and optical properties of PbGa_2_S_4_ under room conditions. From the structural point of view, PbGa_2_S_4_ is a layered material that crystallizes in the orthorhombic EuGa_2_S_4_-type structure (space group No. 70, *D*_2h_^24^−*Fddd*).^2,13–15^ The crystal structure (see Fig. 1 and Fig. S1 and S2 in the ESI†) is built on a framework of GaS_4_ tetrahedral units and square antiprismatic PbS_8_ polyhedra. The GaS_4_ tetrahedra are located in layers stacked along the *c*-axis, where such layers are constructed from edge-shared Ga_2_S_6_ dimers connected to three other dimers *via* sharing corners. By contrast, the PbS_8_ polyhedra are linked to two other PbS_8_ polyhedra by edge-sharing along the *c*-axis and to four other polyhedra by corner-sharing in the *ab*-plane. The presence of the lone electron pair of Pb^2+^ could be related to the crystal structure (S. G. *Fddd*) of PbGa_2_S_4_ in contrast for instance with CdGa_2_S_4_ or HgGa_2_S_4_ with a defect chalcopyrite structure. However, it must be considered that PbGa_2_S_4_ has the orthorhombic EuGa_2_S_4_-type structure also common to CaGa_2_S_4_ and SrGa_2_S_4_. Neither Eu nor Ca or Sr have lone electron pairs, so it is unlikely that the formation of the orthorhombic phase is influenced by the lone electron pair of Pb^2+^. Instead, the large eight-fold coordination for the A cation in the *Fddd* phase seems to be related to the large ionic size of Ca(1.12), Eu(1.25), Sr(1.26), and Pb(1.29).^16^ Curiously, BaGa_2_S_4_, with the Ba atom having a much larger ionic size for eight-fold coordination (1.42), does not crystallize in the *Fddd* structure but in a cubic one described by space group *Pa*3̄.^2^ Note also that the effect of the lone electron pair in Pb when linked to the S atom is very small. In fact, PbS crystallizes in the highly symmetric cubic rock-salt phase while GeS and SnS crystallize in a distorted orthorhombic phase described by space group *Pnma*.

Regarding vibrational properties, phonons from Raman scattering measurements were initially reported by Syrbu *et al.*^17^ and infrared (IR) modes were reported from absorption measurements by Neumann *et al.*^18^ The first work interpreted the Raman modes in the light of a wrong crystal structure, while the latter work interpreted the IR modes in the light of the good crystal structure of PbGa_2_S_4_. Further characterization studies of Raman-active and IR-active modes were also performed, but again were interpreted in the light of the wrong crystal structure.^19,20^ Fortunately, the Raman spectrum at room temperature was well explained in terms of the right crystal structure by Bletskan *et al.*^15^ and more recently by Kamenshchikov *et al.*^21^ Regarding the optical properties, the IR spectral region of PbGa_2_S_4_ was studied,^22^ followed by optical-absorption measurements that determined that it is an indirect bandgap semiconductor, whose indirect bandgap energy, *E*_g_, at room temperature was 2.84 eV, and shows a direct bandgap at 2.91 eV.^23^ Later, Badikov *et al.* reported the refractive index in the visible region and the lasing by doping with Na and Dy atoms.^24^ More recently, the reflectance spectrum was analyzed showing excitons with large binding energy and oscillator strength even at room temperature.^25,26^

The effect of temperature on the properties of PbGa_2_S_4_ has been considerably investigated. Regarding structural properties, no phase change has been observed on decreasing the temperature down to 10 K when measuring different optical properties.^26^ On the other hand, Chilouet *et al.* found a decomposition of PbGa_2_S_4_ above 900 °C^13^ that was recently confirmed.^11^ Moreover, a different polymorph of PbGa_2_S_4_ (S. G. *Pna*2_1_) has been recently synthesized at high temperature.^27^ Regarding vibrational properties, Raman-active phonons were measured at different temperatures between 10 and 300 K.^19,28^ Finally, regarding optical properties, a decrease of the bandgap was reported on increasing the temperature to between 10 and 300 K.^23,29^

Unlike the effect of temperature on the properties of PbGa_2_S_4_, the effect of pressure on the properties of PbGa_2_S_4_, or of any other EuGa_2_S_4_-type compounds, has not been investigated yet, to the best of our knowledge. This contrasts with the properties of A^II^B^III^_2_X^VI^_4_ chalcogenides with spinel, defect chalcopyrite, and their related structures that have been thoroughly studied under compression as recently reviewed.^30^ In this work, we report a joint experimental and theoretical study of the structural, vibrational, and optical properties of PbGa_2_S_4_ under compression of up to 25 GPa. In particular, we report high-pressure (HP) X-ray diffraction (XRD), Raman scattering (RS), and optical-absorption (OA) measurements and compare them to *ab initio* calculations. We show evidence of a partially reversible pressure-induced decomposition of PbGa_2_S_4_ into Pb_6_Ga_10_S_21_ and Ga_2_S_3_ above 15 GPa and provide the equation of state of the low-pressure phase and the pressure dependence of Raman-active modes and of the indirect bandgap of PbGa_2_S_4_ and Pb_6_Ga_10_S_21_. This study allows a comparison to be made for the first time between the properties of EuGa_2_S_4_-type compounds under compression and those of other compounds based on GaS_4_ polyhedra, such as Ga_2_S_3_ and (Zn,Cd,Hg)Ga_2_(S,Se)_4_. Hopefully, this study will also help in understanding the properties of PbGa_2_S_4_ at room conditions in order to improve its technological applications.

## 2 Experimental and theoretical details

### 2.1 Experimental details

PbGa_2_S_4_ crystals have been grown *via* the Bridgman–Stockbarger method in quartz ampoules with high purity (99.999%) source components. The temperature of the melt exceeded the PbGa_2_S_4_ melting temperature (890 °C) by 50–70 °C. The temperature gradient was 20–30 K cm^−1^ at a pull down rate of 0.25 mm h^−1^.^31,32^ High resistivity p-type crystals with sizes of 2 × 2 × 5 cm^3^, which are easily cleaved, were grown and the cleaved surfaces were not mechanically processed.^25^

To be sure about the nature of the as-grown sample, structural characterization of the PbGa_2_S_4_ sample at room pressure (RP) was performed by single-crystal measurements of an orange plate-shaped crystal with dimensions 0.12 × 0.11 × 0.04 mm^3^. Data were collected using a SuperNova, Dualflex, EosS2 diffractometer and measured using *ω* scans with Mo K*α* radiation (*λ* = 0.71073 Å) at a maximum resolution of *Θ* = 28.278° (0.75 Å). The program CrysAlisPro (Rigaku, V1.171.40.84a, 2020) was used to determine the total number of runs and images for the diffraction pattern as well as to index and refine it. A numerical absorption correction based on Gaussian integration over a multifaceted crystal model was performed using spherical harmonics as implemented in the SCALE3 ABSPACK scaling algorithm.

For HP-RS, HP-XRD, and HP-OA measurements in powder or single crystal samples of PbGa_2_S_4_, the samples were placed in a 250 μm-diameter hole in a stainless-steel gasket pre-indented to a thickness of 50 μm inside a membrane-driven diamond-anvil cell (DAC) equipped with diamonds with a culet of 500 μm-diameter. A 4 : 1 methanol–ethanol (M–E) mixture was used as a pressure-transmitting medium (PTM) in all HP experiments. This medium is quasi-hydrostatic up to 10.5 GPa. Special attention was paid to avoid the sample bridging between the diamond anvils.^33^ For measuring the pressure, the ruby fluorescence scale was used.^34^ In addition, Cu grains were loaded close to the sample in the HP-XRD experiments in order to use it as a second pressure scale.^35^

Structural characterization at HP was performed by means of angle-dispersive powder HP-XRD measurements at the BL04-MSPD beamline of the ALBA synchrotron, employing a monochromatic X-ray beam with *λ* = 0.4246 Å focused to 20 × 20 μm^2^ (full width half-maximum).^36^ The X-ray beam was focused by Kirkpatrick–Baez mirrors. XRD images were collected using a Rayonix SX165 CCD detector located 240 mm from the sample. The detector parameters were calibrated using LaB_6_ as the standard. The two dimensional diffraction images were integrated into one-dimensional profiles of intensity *versus* 2*θ* using Dioptas.^37^ XRD profiles were analyzed using GSAS-II by means of Rietveld refinements or by using the Le Bail method.^38^

Vibrational characterization was carried out by means of polarized and unpolarized RS measurements at RP and HP in order to distinguish between the large number of phonons expected for this compound. All RS measurements were carried out with a Horiba Jobin Yvon LabRAM UV HR microspectrometer equipped with a thermoelectrically cooled multichannel CCD detector that allows a spectral resolution better than 2 cm^−1^. The Raman signal was excited with a HeNe laser (632.8 nm line) with a power of less than 10 mW and collected in back-scattering geometry using a ULF notch filter that allows signals to be obtained down to 10 cm^−1^. The frequencies of the Raman-active first-order phonons were obtained after fitting the Raman peaks with Voigt profiles of fixed Gaussian linewidth to the experimental setup resolution (1.6 cm^−1^).

Optical characterization was performed by means of OA experiments at RP and HP in single crystals using two different home-built optical setups that consist of a tungsten lamp, fused silica lenses, reflecting micro-objectives (15×), and a visible-near-IR spectrometer (Ocean Optics Maya2000 Pro in the first setup and Ocean Optics HR2000+ in the second setup). The experimental transmittance of the sample was obtained with the sample-in, sample-out method,^39^ and then scaled to the theoretical transmittance value in the spectral range where the sample is completely transparent. Finally, the absorption coefficient *α* was obtained from the scaled transmittance by taking into account the sample thickness (*d* ≈ 20 μm) and also the reflectivity obtained from the knowledge of the refractive index (*n* ≈ 3.0)^24^ as done in previous works.^40–42^

### 2.2 Theoretical details

*Ab initio* total-energy calculations have been carried out within the density–functional theory (DFT) framework,^43^ using plane waves and the pseudopotential technique with the Vienna *ab initio* simulation package (VASP).^44^ The set of plane waves employed extended up to a kinetic energy cutoff of 400 eV. The generalized gradient approximation (GGA) with the Perdew–Burke–Ernzerhof PBE parametrization was used for the description of the exchange and correlation energy.^45^ Dense special point grids of 4 × 4 × 4 were used to sample the Brillouin zone (BZ) when relaxing the structure at different volumes. Pressure, like other energy derivatives, is obtained simultaneously from the stress tensor.^46^ Lattice dynamics calculations were performed at the zone center (*Γ*-point) of the Brillouin zone. The super-cell method with the primitive cell was employed for the calculation of the dynamical matrix at the *Γ*-point.^44^ Since GGA–PBE calculations underestimate the bandgap values, we have used the metaGGA^47^ approach with the modified Becke–Johnson (MBJ) potential,^48^ which leads to a good agreement with experiments, for electronic band structure calculations.

## 3 Results and discussion

### 3.1 Structural properties

Single-crystal XRD measurements at RP with *R*_1_ = 2.36% confirm that PbGa_2_S_4_ crystallizes in the orthorhombic structure (space group *Fddd*). The unit-cell parameters *a* = 12.1674(2) Å, *b* = 20.4180(4) Å, and *c* = 20.7005(5) Å were refined using 7590 reflections, 31% of the observed reflections. The atomic positions are summarized in Table 1. Further details of the single-crystal XRD analysis are provided in the ESI.† In particular, the final equivalent isotropic displacement parameters, as well as selected bond distances, and angles are provided in Tables S1–S3 in the ESI.† Our structure and values of unit-cell parameters agree with those reported from recent single-crystal XRD measurements.^14^ As already commented, the structure of PbGa_2_S_4_ is composed of a network of GaS_4_ tetrahedra and PbS_8_ polyhedra. The large values of Pb–S distances compared to Ga–S distances are related to the soft ionic Pb–S bonds and the stronger covalent Ga–S bonds and are responsible for the easy cleavage of lead thiogallate into layers that run perpendicular to the longest axis (the *c*-axis in our description).

**Table 1:** Fractional atomic coordinates and isotropic displacement (*U*_eq_) parameters (in Å^2^) for PbGa_2_S_4_ at room conditions. *U*_eq_ is defined as 1/3 of the trace of the orthogonalised *U*_*ij*_

Atom	Wyckoff position	*x*	*y*	*z*	*U*_eq_
Pb3	8a	0.875	0.375	0.375	0.022(12)
Pb2	16g	0.375	0.375	0.127(2)	0.022(11)
Pb1	8b	0.375	0.375	0.375	0.024(13)
Ga1	32h	0.585(4)	0.304(2)	0.249(3)	0.012(12)
Ga2	32h	0.626(4)	0.487(2)	0.250(3)	0.012(12)
S2	32h	0.501(15)	0.250(9)	0.333(5)	0.012(2)
S3	32h	0.748(15)	0.499(9)	0.166(5)	0.012(2)
S4	32h	0.765(8)	0.329(5)	0.248(10)	0.011(2)
S1	32h	0.503(9)	0.406(5)	0.251(11)	0.013(2)

**Fig. 2:**
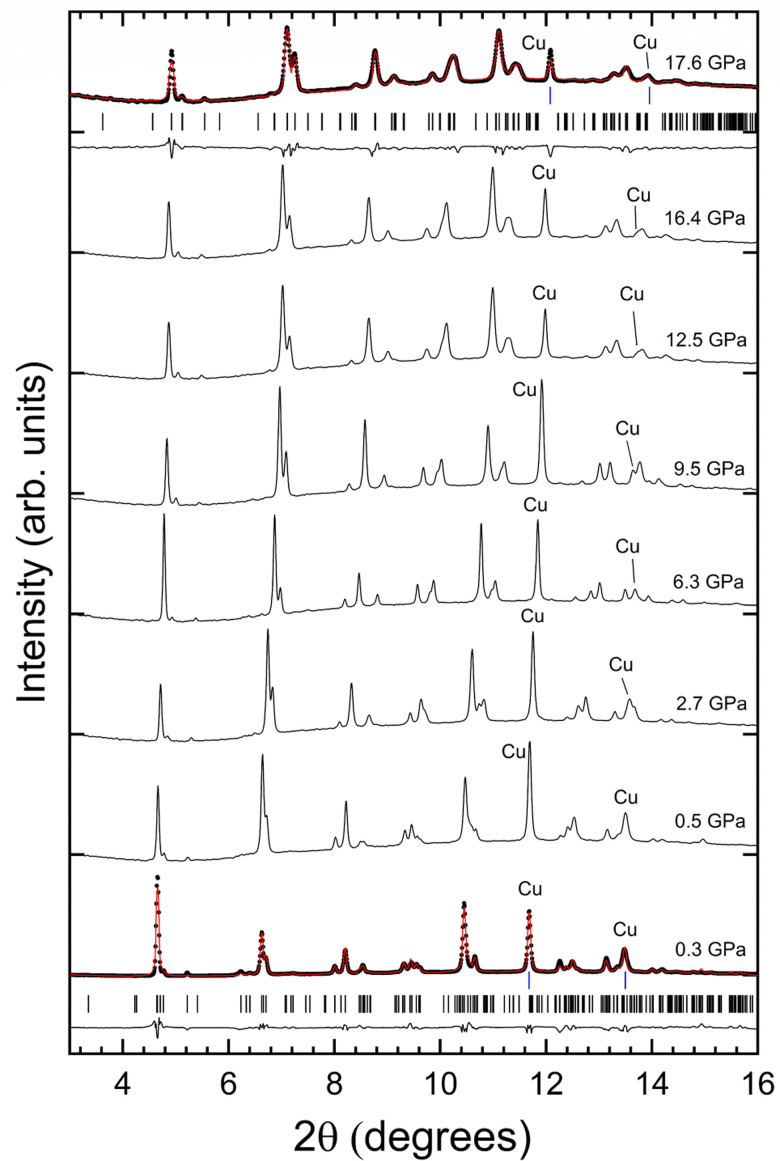
HP-XRD patterns of PbGa_2_S_4_ on compression up to 17.6 GPa. In the bottom and top traces, black dots are the experiments, red lines are the results of structural refinements Rietveld, and black lines are the residuals. The black and blue ticks give the positions of calculated reflections for the sample and Cu, respectively. The pressures are indicated in the figure.

Fig. 2 shows the evolution of the powder XRD patterns of PbGa_2_S_4_ under compression from 0.3 to 17.6 GPa. As pressure increases, the diffraction peaks move gradually to higher angles due to the reduction of unit-cell parameters. Within this pressure range, all peaks can be indexed with an orthorhombic crystal structure isomorphic to the RP phase. The structure at 0.3 GPa is described by space group *Fddd* and the unit-cell parameters are *a* = 12.159(3) Å, *b* = 20.397(6) Å, *c* = 20.67(6) Å. The only difference with the structure at RP is the slight reduction of unit-cell parameters. From 0.3 to 17.6 GPa the sample does not present evidence of any structural phase transition. All the XRD patterns can be explained by the low-pressure orthorhombic structure. This conclusion is supported by Rietveld refinements at the lowest and highest pressures. Notice that the accessible 2*θ* is restricted to 16° (and consequently the number of independent reflections) by the geometrical opening cone of the DAC and the size of the CCD detector. Therefore, the atomic positions were fixed in the refinement at all pressures to the values determined from single-crystal XRD at ambient pressure (see Table 1). In the refinement, we fitted the unit-cell parameters, peak-shape parameters (*U*, *V*, and *W* Caglioti coefficients), the overall displacement factor, and the background (using a 10-term Chebyshev polynomial of the first kind). This approximation usually works quite well for synchrotron HP data.^49^

**Fig. 3:**
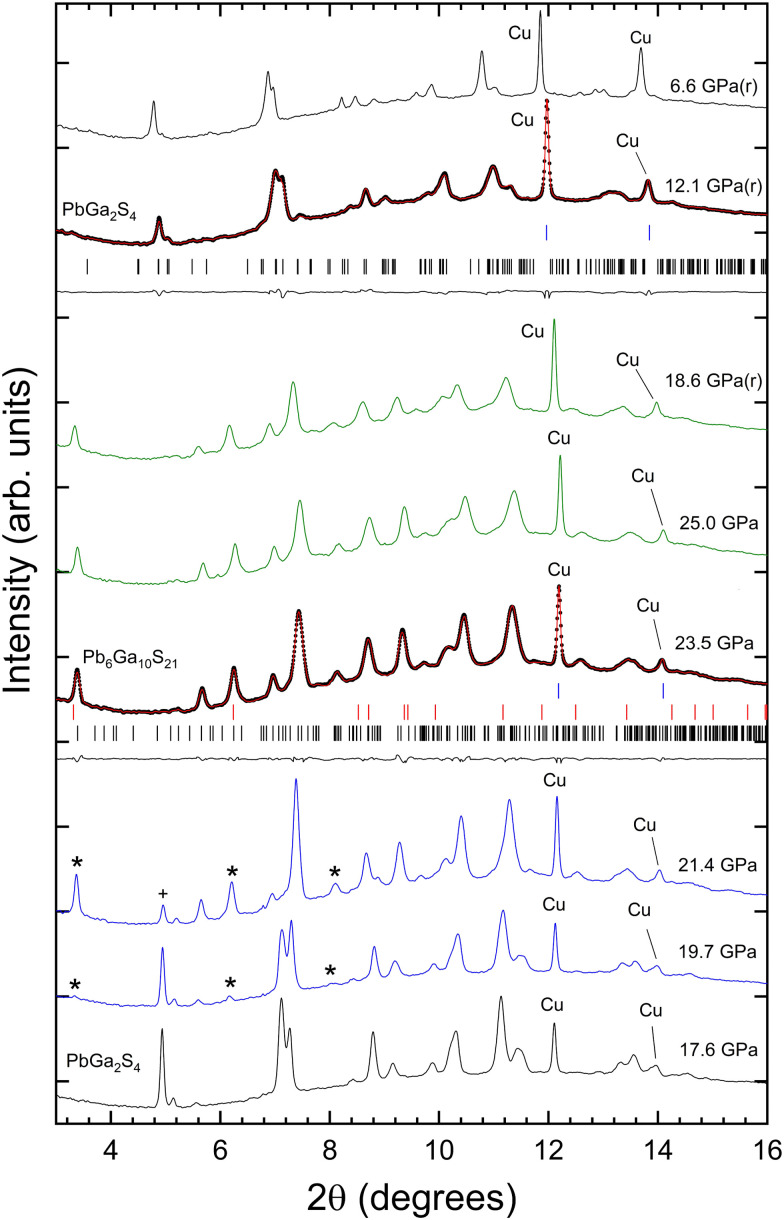
HP-XRD patterns of PbGa_2_S_4_ on compression from 17.6 to 25 GPa. We also include patterns measured upon decompression. These patterns are identified by (r) next to the pressure. We show in blue XRD patterns where the onset of decomposition is detected and in green patterns assigned to Pb_6_Ga_10_S_21_. At 23.6 GPa and 12.1 GPa (r), black dots are the experiments, red lines are the Lebail fits, and black lines are the residuals. The black and blue ticks give the positions of calculated reflections for the sample and Cu, respectively. The red ticks are calculated reflections of Ga_2_S_3_. The pressures are indicated in the figure.

Beyond 17.6 GPa, on the upstroke, remarkable changes can be observed in the XRD patterns (Fig. 3) between 17.6 and 25 GPa. We are aware that at these pressures our PTM is not hydrostatic. However, changes start to develop at 17.6 GPa and the quasi-hydrostatic limit of the PTM is 10.5 GPa. This suggests that the observed structural changes are not related to non-hydrostatic effects. Regarding the observed changes, first, we noticed the emergence of extra peaks, denoted by asterisks in the figure, and the weakening of peaks assigned to the RP phase, denoted by the plus symbol in the figure. The patterns measured at 19.7 and 21.4 GPa correspond to the coexistence of the RP and HP phases. At 23.5 and 25 GPa the patterns can be identified with only the HP phase. The DICVOL routine was used to index the Bragg reflections of the HP phase measured at 23.5 GPa and CheckCell was used for space group determination. For these purposes, we used only the peaks below 2*θ* = 12° to avoid the overlapping of reflections from the HP phase and Cu. It was found that the monoclinic space group *C*2/*m* gives the best figure of merit; *M*(20) = 23.2. A subsequent Le Bail fit, using the structural model obtained from the method described above, provided as unit-cell parameters *a* = 25.269(9) Å, *b* = 3.529(2) Å, *c* = 14.433(5) Å, and *β* = 96.632°. Fig. 3 shows the good profile match obtained between the Le Bail fit and the measured XRD pattern.

**Fig. 4:**
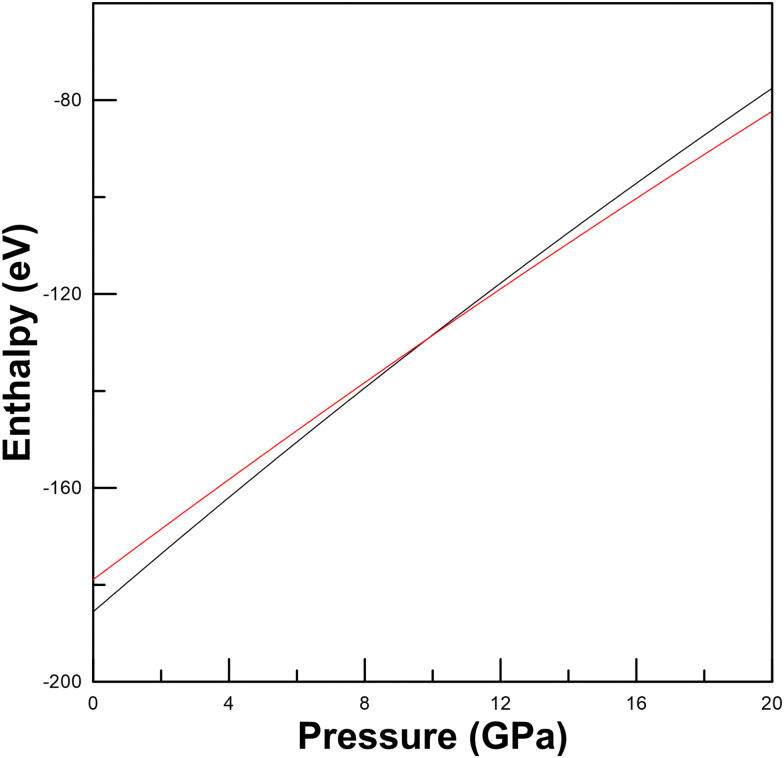
Calculated enthalpy of the orthorhombic structure of PbGa_2_S_4_ (black solid line) and the sum of the enthalpy of decomposition products Pb_6_Ga_10_S_21_ and Ga_2_S_3_ (red solid line).

Interestingly, the space group and unit-cell parameters obtained for the X-ray diffraction patterns of the HP phase above 21.4 GPa are similar to those of Pb_6_In_10_S_21_.^50^ Therefore, we performed theoretical calculations on Pb_6_Ga_10_S_21_ at different pressures. For calculations, we have used both the RP phase of Ga_2_S_3_, α′-Ga_2_S_3_, with a monoclinic *Cc* structure,^51^ and the HP phase above 16 GPa, β′-Ga_2_S_3_, with a rhombohedral *R*3̄*m* structure.^52^ Our enthalpy *vs.* pressure calculations showed that it is thermodynamically favorable for PbGa_2_S_4_ to decompose into Pb_6_Ga_10_S_21_ + Ga_2_S_3_ above 10 GPa (see Fig. 4) since the enthalpy of the decomposition products becomes smaller than that of the father compound beyond this pressure. In fact, the decomposition process is 6 formula units of PbGa_2_S_4_ decompose into Pb_6_Ga_10_S_21_ + Ga_2_S_3_. Consequently, we think that both our XRD experiments and calculations support the decomposition hypothesis.

**Fig. 5:**
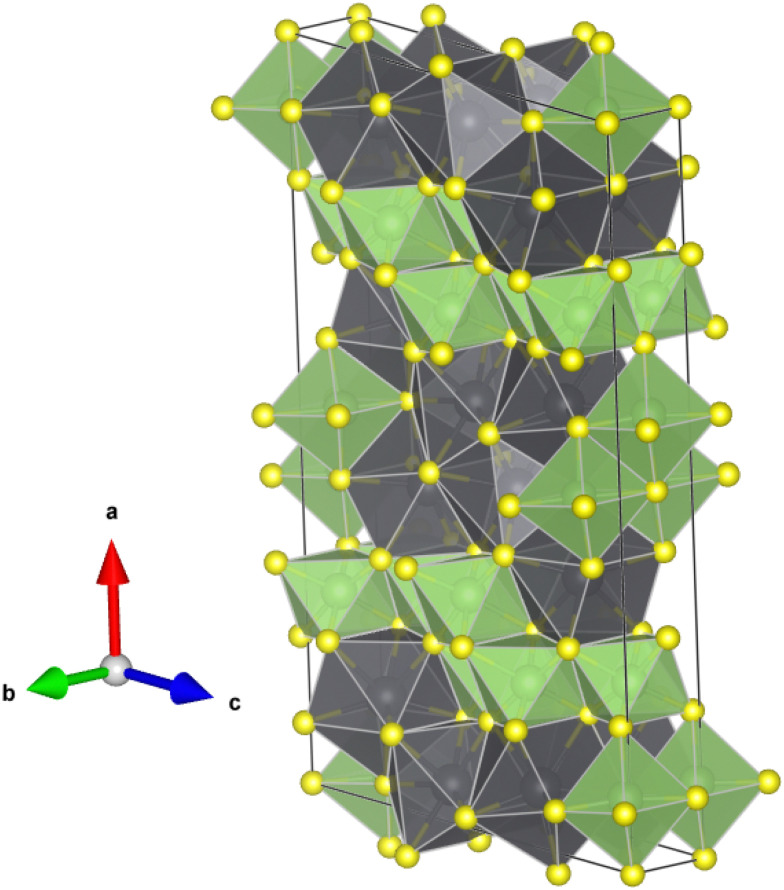
Schematic representation of the crystal structure of Pb_6_Ga_10_S_21_. GaS_6_ octahedral units are shown in green and PbS_8_ bicapped trigonal prismatic polyhedra in black.

Unfortunately, the XRD peaks of Ga_2_S_3_ are much weaker than those of Pb_6_Ga_10_S_21_ (because of the strong X-ray absorption of Pb) and they overlap with those of Pb_6_Ga_10_S_21_ (see Fig. 3). Therefore, the XRD patterns of the HP phase have been explained by a Le Bail fit and not by a Rietveld refinement. In any case, the calculated lattice parameters at 23.5 GPa (*a* = 25.3061 Å; *b* = 3.5276 Å; *c* = 14.4180 Å; and *β* = 96.7820°) agree with the experimental values we reported in the previous paragraph what gives further support to the decomposition process already commented. For completeness, the theoretical atomic positions of Pb_6_Ga_10_S_21_ at 23.5 GPa are reported in Table 2. The crystal structure of Pb_6_Ga_10_S_21_ is shown in Fig. 5. In contrast with the studied compound, this material is not layered, the crystal structure being formed by zig-zag chains of edge sharing GaS_6_ octahedra and PbS_8_ dodecahedra. In summary, we conclude with confidence that PbGa_2_S_4_ undergoes a pressure-induced decomposition at HP. This observation is not fully unexpected since it has been recently found that this compound tends to decompose into PbS and Ga_2_S_3_ during the growth process of single crystals if temperature is larger than 1250 K.^53^

**Table 2:** Fractional atomic coordinates of Pb_6_Ga_10_S_21_ at 23.5 GPa. They have been obtained from DFT calculations using the PBE functional

Atom	Wyckoff position	*x*	*y*	*z*
S1	2a	0.0000	0.0000	0.0000
S2	4i	0.9728	0.0000	0.7662
S3	4i	0.6730	0.0000	0.7606
S4	4i	0.7647	0.0000	0.6006
S5	4i	0.5798	0.0000	0.8778
S6	4i	0.3975	0.0000	0.9046
S7	4i	0.3796	0.0000	0.6297
S8	4i	0.5373	0.0000	0.5955
S9	4i	0.1934	0.0000	0.9776
S10	4i	0.2790	0.0000	0.8218
S11	4i	0.1511	0.0000	0.5670
Ga1	4i	0.3121	0.0000	0.5110
Ga2	4i	0.2174	0.0000	0.6926
Ga3	4i	0.7323	0.0000	0.8966
Ga4	4i	0.4863	0.0000	0.8711
Ga5	4i	0.0983	0.0000	0.9850
Pb1	4i	0.0807	0.0000	0.7200
Pb2	4i	0.9493	0.0000	0.5615
Pb3	4i	0.8565	0.0000	0.7601

Under decompression, Pb_6_Ga_10_S_21_ is still observed at 18.6 GPa; however, the RP phase is recovered at 12.1 GPa and it is also observed at 6.6 GPa. The peaks of the RP phase after decompression are slightly broader that those measured at similar pressures upon compression due to residual stresses in already compressed samples. The reversibility of changes induced by pressure indicates that, in an unexpected way, the pressure-induced decomposition is reversible after pressure release; a fact rather uncommon but that has been previously reported in other compounds.^54^ In our opinion, the reversibility of the pressure-induced decomposition in PbGa_2_S_4_ is favored because 6 formula units of PbGa_2_S_4_ (Pb_6_Ga_12_S_24_) decompose into Pb_6_Ga_10_S_21_ + β′-Ga_2_S_3_. Therefore, on decompression Pb_6_Ga_10_S_21_ can transform back into 5 PbGa_2_S_4_ + PbS, thus resulting a mixture of 5 PbGa_2_S_4_ + PbS + γ-Ga_2_S_3_ at room conditions from the original 6 formula units of PbGa_2_S_4_. Note that the RP phase α′-Ga_2_S_3_, with monoclinic *Cc* structure, transforms into β′-Ga_2_S_3_, with rhombohedral *R*3̄*m* structure^52^ above 16 GPa and on decompression to room-pressure the original structure of Ga_2_S_3_ is not recovered and a γ phase is observed instead.^52^

**Fig. 6:**
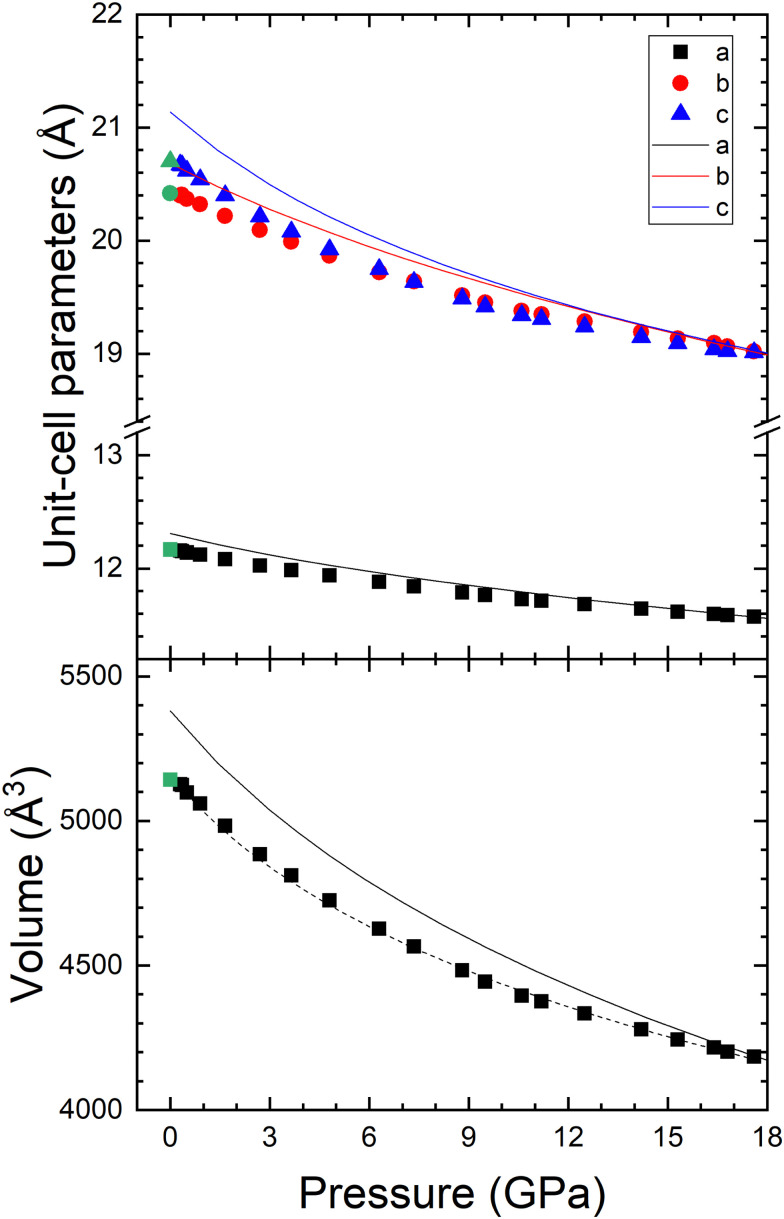
Pressure dependence of the lattice parameters and volume in PbGa_2_S_4_. Symbols represent the experiments, lines represent the calculations and dashed lines represent the equations of states determined from experiments. The same color is used for the same parameter in experimental and theoretical results. Green symbols represent the experiment at ambient pressure.

Let us now analyse the evolution of the structure of the RP phase of PbGa_2_S_4_. A good agreement has been found between the experimental and theoretical pressure dependence of the unit-cell parameters and unit-cell volume of PbGa_2_S_4_ (see Fig. 6). Curiously, the *b*- and *c*-axes tend to become equal at HP, thus indicating and enhancement of the symmetry of the crystal structure upon compression. However, the structure cannot be described as tetragonal above 12 GPa because XRD patterns cannot be indexed by any space group with a fourfold rotation axis. The experimental axial compressibilities have been determined by the following expression 
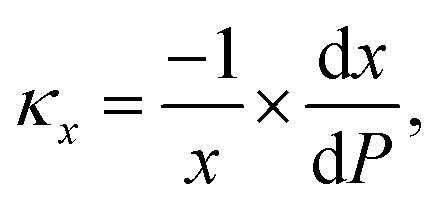
 and are *κ*_a_ = 4.33 × 10^−3^ GPa^−1^, *κ*_b_ = 6.69 × 10^−3^ GPa^−1^, and *κ*_c_ = 8.12 × 10^−3^ GPa^−1^. As a result, there is a considerable anisotropic compression of PbGa_2_S_4_ since the *b*- and *c*-axes are more compressible than the *a*-axis.

From the experimental and theoretical data given in Fig. 6, the pressure–volume (*P*–*V*) equation of state (EoS) for the orthorhombic phase has been obtained with a third-order Birch–Murnaghan EoS (BM3-EOS),^57^ using the software EosFit.^58^ The zero-pressure unit-cell, *V*_0_, bulk modulus, *B*_0_, and its pressure derivative, 
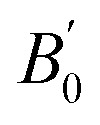
 are summarized in Table 3. It can be observed that the experimental and theoretical results are in relatively good agreement. For comparison purposes, Table 3 also shows the corresponding experimental parameters obtained in other AGa_2_S_4_ compounds. Since both parameters are correlated^59^ and they may depend on experimental conditions,^60^ the pressure-transmitting medium (PTM) used in each experiment is also reported in Table 3. It can be observed that for PbGa_2_S_4_ the experimental *B*_0_ (47.3(1) GPa) obtained with the BM3-EOS is similar to that obtained for HgGa_2_S_4_, but the bulk modulus is 40% smaller than in CdGa_2_S_4_. The reason for the larger bulk modulus of CdGa_2_S_4_ could be the poorer hydrostatic conditions of the experiments performed in this compound, as discussed by Errandonea *et al.*^61^ This hypothesis is consistent with the fact that DFT calculations give for CdGa_2_S_4_ a bulk modulus of 40.8–46.0 GPa.^62,63^ In any case, a slightly smaller value of *B*_0_ is expected for EuGa_2_S_4_-type compounds than for defect chalcopyrite AGa_2_S_4_ (A = Cd, Hg) compounds since a larger compressibility is expected for eightfold-coordinated Pb atoms in the orthorhombic *Fddd* structure than for fourfold-coordinated Cd and Hg atoms in the defect chalcopyrite structure described by the space group *I*4̄.

**Table 3:** EoS parameters (*V*_0_, in Å^3^, *B*_0_, in GPa, and 
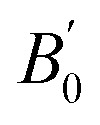
 dimensionless) for several AGa_2_S_4_ compounds. The pressure-transmitting medium used in experiments is indicated

Compound	*V*_0_	*B*_0_	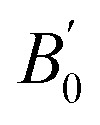	PTM	Ref.
PbGa_2_S_4_	5142(1)	47.3(1)	4.4(3)	Methanol–ethanol	This work – experiment
PbGa_2_S_4_	5380	39	4.7		This work – theory
CdGa_2_S_4_	311.4(9)	64(2)	4.1(3)	Silicone oil	55
HgGa_2_S_4_	309.80(14)	48.1(9)	4.1(3)	Methanol–ethanol	56

Curiously, the experimental and theoretical bulk moduli of PbGa_2_S_4_ are also similar to those of the RP phase of Ga_2_S_3_ (α′-Ga_2_S_3_, experimental *B*_0_ = 47 GPa and theoretical *B*_0_ = 38 GPa). In fact, also the axial compressibilities of monoclinic α′-Ga_2_S_3_ are also similar to those of PbGa_2_S_4_.^51^ Noteworthy, α′-Ga_2_S_3_ is composed of GaS_4_ tetrahedra with 1/3 empty cation sites and it was shown that the two GaS_4_ tetrahedra in α′-Ga_2_S_3_ feature a much higher polyhedral bulk modulus (99.1 and 123.0 GPa) than the compound bulk modulus. On the basis of this data, we expect a similar polyhedral bulk modulus for the GaS_4_ tetrahedra in PbGa_2_S_4_ and other AGa_2_S_4_ compounds. Consequently, our results suggest that the bulk modulus of PbGa_2_S_4_ is mainly determined by the compressibility of the PbS_8_ polyhedra and that, surprisingly, they compress at a similar rate than cation vacant sites in Ga_2_S_3_, what is in agreement with the weak Pb–S bonds and easy cleavage of layers in PbGa_2_S_4_.

### 3.2 Vibrational properties

The orthorhombic *Fddd* structure of PbGa_2_S_4_ at RP contains 32 formula units in the unit cell (224 atoms) and its primitive unit cell contains 8 formula units (56 atoms). Therefore, according to group theory, PbGa_2_S_4_ should have 168 vibrational modes at the *Γ* point with the mechanical representation:^64^*Γ* = 19A_g_ + 19A_u_ + 21B_1g_ + 21B_1u_ + 22B_2g_ + 22B_2u_ + 22B_3g_ + 22B_3u_From the 168 vibrational modes, there are 165 optical modes and 3 acoustic modes (B_1u_ + B_2u_ + B_3u_). Since the structure is centrosymmetric, there is a separation between Raman-active (gerade, g) and IR-active (ungerade, u) modes. Therefore, there is a total of 84 Raman-active and 81 IR-active first-order modes.

Due to the large number of Raman-active modes allowed in PbGa_2_S_4_, polarised and unpolarised RS measurements were performed in order to better distinguish their symmetry. From the polarized measurements at RP, we found the 6 different types of symmetry, A_g_ (*xx*), A_g_ (*zz*), A_g_ (*yy*), B_1g_ (*xz*), B_2g_ (*xz*) and B_3g_ (*xz*) (see Fig. S3 in the ESI†), that are in good agreement with those already published by Kamenshchikov *et al.*^21^ A comparison of the frequencies of the different modes observed at room conditions in this work and by Kamenshchikov *et al.* is reported in Table S4 in the ESI.†

The Raman spectrum of PbGa_2_S_4_ can be divided into two regions: the low- (high-) frequency region below (above) 200 cm^−1^. Our calculations confirm that modes of the high-frequency region are related to the internal Ga–S stretching modes of the GaS_4_ tetrahedra, while modes of the low-frequency region are related to Ga–S bending modes of the GaS_4_ tetrahedra (between 100 and 200 cm^−1^) and to modes related to Pb vibrations as well as to rigid translations and rotations of GaS_4_ tetrahedra (below 100 cm^−1^). In general, the modes related to Pb vibrations show a smaller intensity and a narrower linewidth than those corresponding to internal (Ga–S bending and stretching) modes of the GaS_4_ tetrahedra. This classification of vibrational modes in PbGa_2_S_4_ according to internal and external modes of GaS_4_ tetrahedra agrees with the one performed in α′-Ga_2_S_3_, where a separation between low- and high-frequency regions is observed below and above 200 cm^−1^.^51^ In particular, the study of the vibrational properties of α′-Ga_2_S_3_ showed that all internal modes of GaS_4_ tetrahedra are above 200 cm^−1^ and that all external modes of GaS_4_ tetrahedra are between 70 and 200 cm^−1^. Similarly, the vibrational modes in CdGa_2_S_4_ (HgGa_2_S_4_) extend from 80 (60) to 400 cm^−1^.^62,65^ Therefore, we can conclude that since Pb has a similar mass as Hg, the existence of vibrational modes below 60 cm^−1^ in PbGa_2_S_4_ at RP must be exclusively ascribed to the vibrations of Pb atoms. This conclusion agrees with the comments of Kamenshchikov *et al.* that attributed the modes related to Pb–S bonds to those observed between 20 and 55 cm^−1^ (note that these authors did not report modes below 20 cm^−1^).^21^

**Fig. 7:**
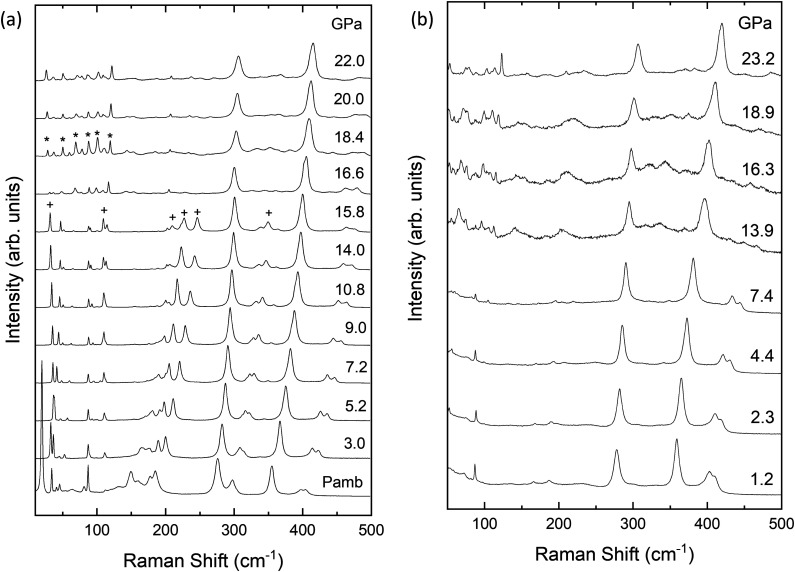
Normalized Raman spectra of PbGa_2_S_4_ at high pressure up to 22 GPa. (a) Upstroke, (b) downstroke. Crosses indicate the main peaks of the *Fddd* phase that disappear with increasing pressure. Stars indicate new peaks of the high-pressure phase.

Regarding the pressure dependence of the Raman-active modes in PbGa_2_S_4_, Fig. 7 shows a selection of unpolarized HP-RS spectra of PbGa_2_S_4_ under compression up to 22 GPa and decompression down to RP. Since samples are exfoliated perpendicular to the *c*-axis, our Raman measurements obtained in back-scattering geometry inside the DAC correspond to a mixture of modes with A_g_, B_2g_ and B_3g_ symmetry. This information has been obtained from our polarized RS measurements at room conditions (see Fig. S4 in the ESI†) and also from HP-RS measurements in which only modes of A_g_ symmetry or a mixture of modes of B_2g_ and B_3g_ symmetry have been distinguished (see Fig. S3 in the ESI†).

Raman spectra of Fig. 7(a) and Fig. S4 (ESI†) show that some of the Raman modes of the RP phase (marked with crosses in Fig. 7) disappear above 16.6 GPa together with the appearance of some extra peaks (marked with asterisks). The changes observed in HP-RS measurements above 16 GPa agree with those from HP-XRD measurements above 17 GPa, thus suggesting the occurrence of a phase transition above 16 GPa. On downstroke, Fig. 7(b) shows similar Raman spectra to those of the RP phase, but with peaks of smaller intensity and larger linewidth, appear below 7.4 GPa, thus supporting the hypothesis that the sample partially reverses to the original RP phase on downstroke from 25 GPa.

**Fig. 8:**
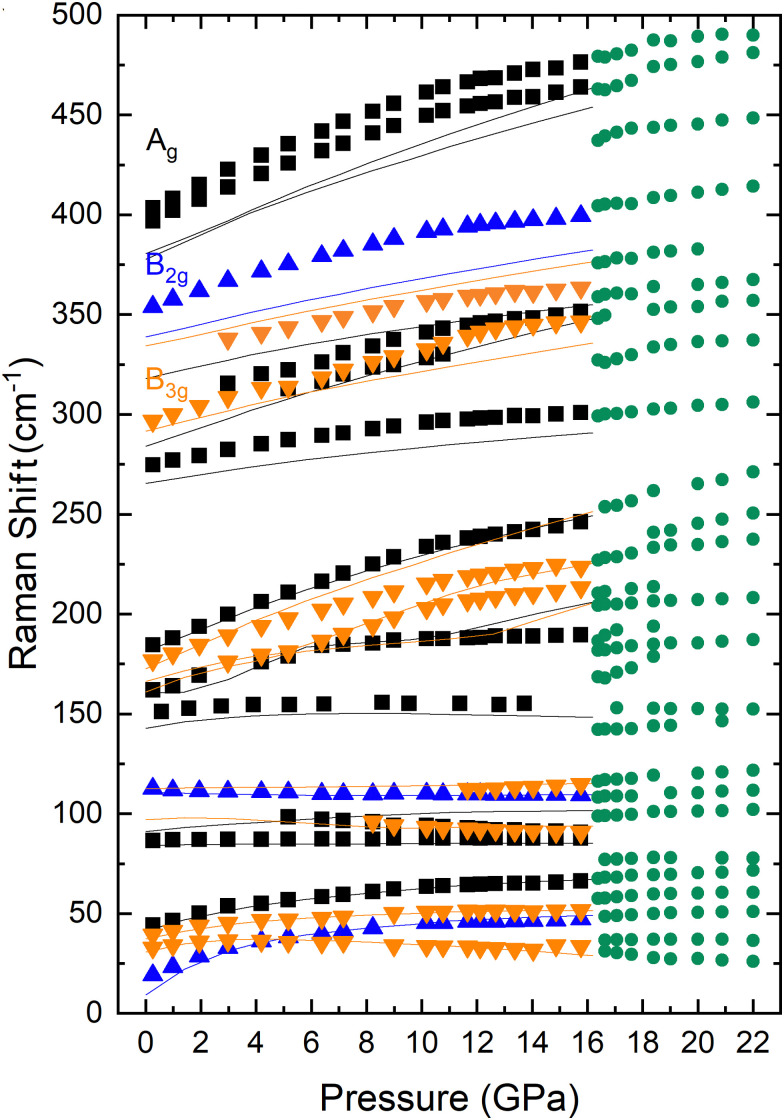
Pressure dependence of the experimental (symbols) and theoretical (lines) Raman frequencies of PbGa_2_S_4_ during compression. We use black color for A_g_ modes, blue color for B_2g_ modes, orange color for B_3g_ modes, and green color for the mode of the HP phase.

From the analysis of the unpolarized HP-RS spectra of PbGa_2_S_4_, 21 of the 84 modes of the compound were distinguished and followed under pressure. The symmetry of those modes was tentatively attributed thanks to the reasonable comparison of the pressure dependence of the experimental and theoretical frequencies (also reported in Fig. 8). The experimentally observed Raman modes and their tentatively assigned theoretical symmetries with their zero-pressure frequencies and pressure coefficients are summarized in Table 4. For the sake of completeness, the zero-pressure frequencies and pressure coefficients of all the theoretically predicted Raman-active modes are summarized in Table S5 in the ESI.†

**Table 4:** Theoretical (th.) and experimental (exp.) Raman-active frequencies at zero pressure (*ω*_0_, in cm^−1^) and pressure coefficients (*a*_1_, in cm^−1^ GPa^−1^; *a*_2_, in cm^−1^ GPa^−2^) in PbGa_2_S_4_ according to fits to *ω*_0_ + *a*_1_*P* + *a*_2_*P*^2^. Theoretically unobserved modes are found in the ESI, Table S5

Mode	PbGa_2_S_4_ th.			PbGa_2_S_4_ exp.		
	*ω*_0_	*a*_1_	*a*_2_	*ω*_0_	*a*_1_	*a*_2_
B^1^_2g_	17.7(2)	3.81(0)	−0.1(0)	20.3(1)	4.1(0)	0.0(0)
B^1^_3g_	33.8(1)	0.7(0)	−0.1(0)	34.7(1)	0.2(0)	0.0(0)
B^2^_3g_	39.2(0)	1.6(0)	−0.1(0)	40.8(0)	1.5(0)	−0.1(0)
A^1^_g_	44.0(1)	2.4(0)	−0.1(0)	45.2(0)	2.7(0)	−0.1(0)
A^2^_g_	84.3(0)	0.1(0)	0.0(0)	86.6(0)	0.2(0)	0.0(0)
A^3^_g_	88.7(1)	1.1(0)	0.0(0)	104.7(1)	−1.4(0)	0.0(0)
B^7^_3g_	108.3(0)	−0.4(0)	0.0(0)	109.9(2)	−2.3(0)	−0.1(0)
B^6^_2g_	110.4(0)	−0.7(0)	0.0(0)	112.3(0)	−0.4(0)	0.0(0)
B^8^_3g_	112.7(0)	0.0(0)	0.0(0)	125.6(4)	−2.4(1)	0.1(0)
A^5^_g_	145.4(1)	0.8(0)	0.0(0)	150.5 (0)	1.6(0)	−0.2(0)
B^10^_3g_	153.5(2)	3.7(0)	−0.1(0)	152.6(1)	3.9(0)	−0.1(0)
A^8^_g_	160.2(1)	3.2(2)	0.0(0)	160.9(1)	4.4(0)	−0.1(0)
B^12^_3g_	162.8(1)	4.7(0)	0.0(0)	158.1(2)	5.6(1)	−0.1(0)
B^14^_3g_	173.9(0)	5.8(0)	0.0(0)	174.9(0)	5.1(0)	−0.1(0)
A^10^_g_	182.3(0)	5.4(0)	0.0(0)	181.8(1)	6.6(0)	−0.2(0)
A^14^_g_	283.3(0)	5.1(0)	−0.1(0)	289.9(4)	5.0(1)	−0.1(0)
B^18^_3g_	292.7(0)	3.1(0)	0.0(0)	295.5(1)	4.2(0)	−0.1(0)
A^15^_g_	320.0(1)	2.4(0)	0.0(0)	299.6(1)	5.3(0)	−0.1(0)
B^19^_3g_	334.4(0)	3.0(0)	0.0(0)	325.6(1)	4.1(0)	−0.1(0)
B^19^_2g_	339.1(0)	3.2(0)	0.0(0)	352.4(0)	5.2(0)	−0.1(0)
A^18^_g_	379.2(0)	5.6(0)	−0.1(0)	394.7(0)	7.0(0)	−0.2(0)
A^19^_g_	380.0(0)	6.1(0)	−0.1(0)	400.8(0)	7.8(0)	−0.2(0)

Regarding the pressure dependence of the Raman-active modes, the modes above 250 cm^−1^ show pressure coefficients similar to those of CdGa_2_S_4_, HgGa_2_S_4_, and α′-Ga_2_S_3_ above 200 cm^−1^.^51,62,65^ In fact, the modes with the largest pressure coefficient are those close to 180 cm^−1^ in PbGa_2_S_4_, and at slightly larger frequencies between 200 and 250 cm^−1^ in the other three compounds. Similarly, modes below 150 cm^−1^ in PbGa_2_S_4_ show very small or even negative pressure coefficients in the same way as modes below 180 cm^−1^ in the other three compounds. Therefore, there is a clear correspondence between the vibrational modes in PbGa_2_S_4_ and in other thiogallates.

Above 16 GPa, new Raman modes were observed together with modes that seem to come from the original RP phase. The pressure dependence of the frequencies of all modes measured above 16 GPa are shown in Fig. 8 and also in more detail in Fig. S5 in the ESI.† Assuming that most of the Raman-active modes observed above 16 GPa correspond to monoclinic Pb_6_Ga_10_S_21_, we have calculated the vibrational modes of this compound at different pressure between 16 and 23 GPa. According to group theory,^64^ Pb_6_Ga_10_S_21_ should have 111 vibrational modes at *Γ* with the mechanical representation:*Γ* = 36A_g_ + 19A_u_ + 18B_g_ + 38B_u_From the 111 vibrational modes, there are 108 optical modes and 3 acoustic modes (A_u_ + 2B_u_). Since the structure is centrosymmetric, there is a separation between Raman-active (gerade, g) and IR-active (ungerade, u) modes. Therefore, there is a total of 54 Raman-active (36A_g_ + 18B_g_) and 54 IR-active (18A_u_ + 36B_u_) modes.

The theoretical frequencies and pressure coefficients of Pb_6_Ga_10_S_21_ at 16 GPa are summarized in Table S6 and plotted in Fig. S5 (ESI†) for their comparison with experimental modes. As can be observed, there is no match between the experimental and theoretical modes since theoretical modes of Pb_6_Ga_10_S_21_ between 16 and 23 GPa are distributed in a smaller frequency range (between 40 and 410 cm^−1^) as compared to experimental modes (between 20 and 470 cm^−1^). The reason for the smaller high-frequency modes in Pb_6_Ga_10_S_21_ compared to PbGa_2_S_4_ is because Ga attains a sixfold coordination in the former compound while it has fourfold coordination in the latter. The larger coordination of Ga in Pb_6_Ga_10_S_21_ leads to larger Ga–S bond distances and smaller stretching frequencies. Note that a similar decrease of stretching frequencies occurs upon the transformation of α′-Ga_2_S_3_ to β′-Ga_2_S_3_.^51,52^ Therefore, we think that modes above 16 GPa are likely due to a mixture of modes of the original PbGa_2_S_4_ compound and of the new Pb_6_Ga_10_S_21_ compound.

### 3.3 Optical properties

**Fig. 9:**
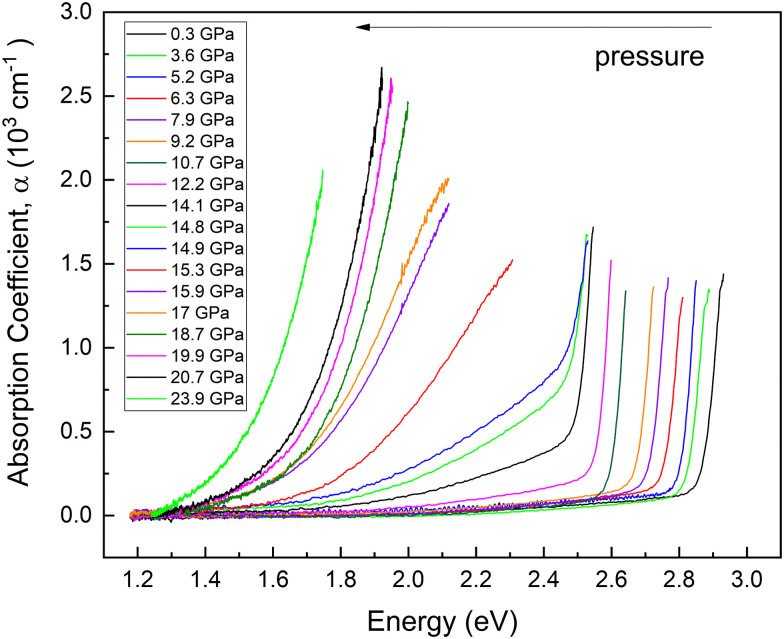
Optical absorption spectra measured at different pressures. Each pressure is shown in a different color. Pressures are indicated in the inset.

HP-OA measurements showing OA spectra with unpolarized light perpendicular to the *c*-axis of the sample are plotted in Fig. 9 at selected pressures up to 23.9 GPa. At RP, an abrupt absorption edge near 2.9 eV is observed. The OA spectra and the value of the bandgap energy were obtained from the original transmittance spectra in the same way as that reported in ref. 23. We found that the fundamental absorption edge energy (2.85 eV) extrapolated from the (*α* × *hν*)^1/2^*vs. hν* curves to the abscissa (Tauc plot) at RP corresponds to the indirect bandgap energy. Both the energy and character of the fundamental absorption edge we have obtained agree with the data previously reported (2.84 eV) by Neumann *et al.* at RP.^23^

As pressure increases the bandgap red shifts and develops a low-energy tail above 14 GPa. In addition, we observed an abrupt shift of the OA edge towards low energies when pressure is increased from 14.9 to 15.3 GPa. The changes in this pressure range are consistent with changes observed in HP-XRD and HP-RS measurements at similar pressures and can be attributed to the already commented pressure-induced decomposition of PbGa_2_S_4_. In fact, we consider that the appearance of the low-energy tail in the OA spectra above 14 GPa is likely due to the creation of defects in the crystal that favour the transmission of diffuse light and/or to the coexistence of the RP phase of PbGa_2_S_4_ and the HP phase corresponding to Pb_6_Ga_10_S_21_.

**Fig. 10:**
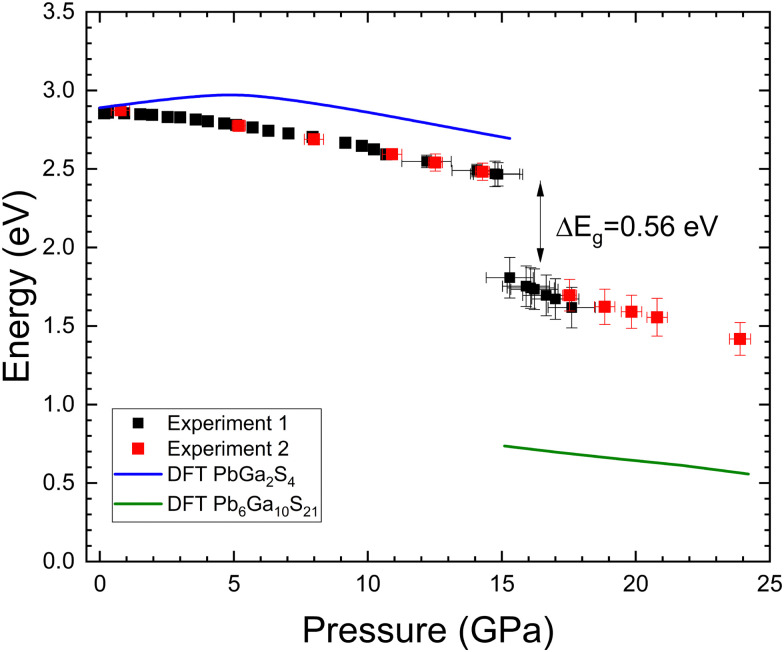
Pressure dependence of the indirect band-gap energy in both compounds as measured from two experiments (shown with black and red dots). The solid line represents the results of calculations. At 15 GPa there is an abrupt decrease of the bandgap of 0.56 eV associated with the observed pressure-induced decomposition of PbGa_2_S_4_.

In Fig. 10 we report the pressure dependence of the bandgap energy obtained from two HP-OA measurements by fitting the high-energy part of the OA spectra with the Tauc plot.^66^ In both experiments, we observed a non-linear pressure dependence which is followed by a collapse of the indirect bandgap of nearly 0.55 eV at the pressure where PbGa_2_S_4_ undergoes the pressure-driven decomposition. In the pressure range of stability of PbGa_2_S_4_, we found that the bandgap first slightly opens and then closes under compression. This behavior agrees with the theoretical pressure dependence of the optical bandgap from our DFT calculations. In this context, our metaGGA calculations predict indirect and direct bandgap energies of 2.89 and 3.00 eV at RP for PbGa_2_S_4_; *i.e.* the calculated bandgap is in good agreement with experiments. In contrast, our PBEsol calculations underestimated both bandgaps by *ca.* 0.32 eV. Calculations also support that the optical bandgap of PbGa_2_S_4_ first increases and then decreases with increasing pressure following a qualitatively similar dependence than observed in experiments.

**Fig. 11:**
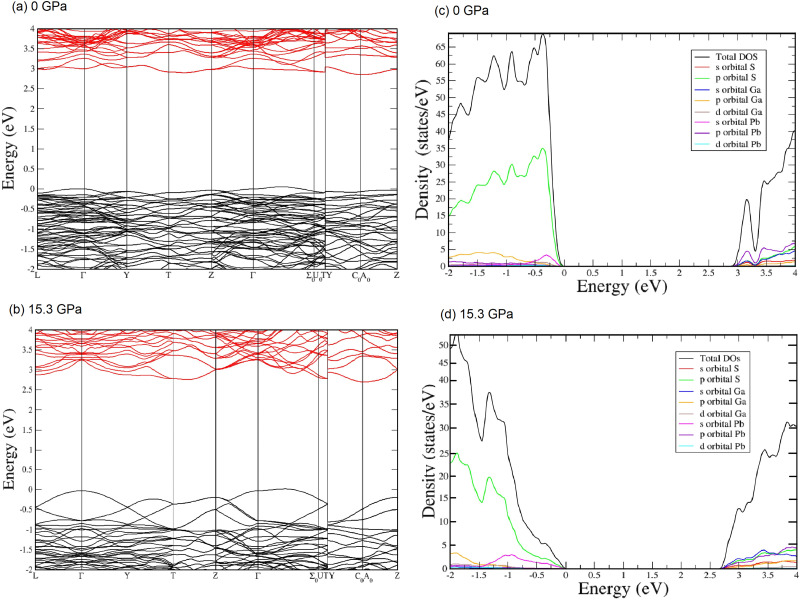
(a) Band-structure at 0 GPa. (b) Band-structure at 15.3 GPa. (c) Total and partial electronic density of states at 0 GPa (d) Total and partial electronic density of states at 15.3 GPa. In Fig. S6 in the ESI,† we zoom on the partial density of states for density values inferior to 10 states per eV.

Fig. 11 shows the electronic band structure and electronic density of states in PbGa_2_S_4_ at 0 and 15.3 GPa. At 0 GPa, both the valence and conduction band are not very dispersive. The bottom of the conduction band is at the *C*_0_/*A*_0_ point of the Brillouin zone and the top of the valence band is between the *Γ* and *Σ*_0_ points of the Brillouin zone. Therefore, our calculations confirm that PbGa_2_S_4_ is an indirect-gap semiconductor. This is not surprising because this compound crystallizes in a space group which is centrosymmetric. In centrosymmetric crystals, no p–d mixing takes place at the *Γ* point, but it does in less symmetrical points leading to upwards (downwards) dispersion in the valence (conduction) band when moving away from the *Γ* point. At 15.3 GPa, the situation is similar to that of 0 GPa (the points where there is the minimum of the conduction band and the maximum of the valence band are the same), but there is a much higher dispersion of the electronic bands.

Regarding the electronic density of states at both 0 and 15.3 GPa, we can observe that the top of the valence band is basically contributed by S 3p states, which are slightly hybridized with Pb 6s states. On the other hand, the bottom of the conduction band is dominated by Pb 6p orbitals. This is the main difference between PbGa_2_S_4_ on one hand and CdGa_2_S_4_ and HgGa_2_S_4_ on the other hand. In the latter compounds, the states near the Fermi level in both the valence and conduction bands are dominated by Ga and S orbitals with no contribution from the divalent cations. Therefore, our calculations show that the interpretation of the assumed similarity of the electronic band structure of Cd and Pb thiogallates made by Neumann *et al.* is not correct.

The difference in orbital contribution to states near the Fermi level between the three thiogallates makes the bandgap of PbGa_2_S_4_ much smaller than that of the other two thiogallates.^67^ In CdGa_2_S_4_ and HgGa_2_S_4_, the increase of the crystal field under compression cause the bandgap to open,^68,69^ and the same occurs in α′-Ga_2_S_3_.^51^ In PbGa_2_S_4_, the contribution of Pb 6s states to the top of the valence band is enhanced when pressure increases. The same happens to the contribution of Ga 3s states to the bottom of the conduction band. Since the increase of the energy of the top of the valence band with pressure in PbGa_2_S_4_ is larger than the increase of the energy of the bottom of the conduction band due to the larger compressibility of the Pb–S bonds in PbS_8_ units than of the Ga–S bonds in GaS_4_ units, after an initial opening, both the indirect and direct bandgaps close as indicated in Fig. 11 and in agreement with our HP-OA measurements once the underestimation of the calculated indirect bandgap is corrected. In summary, the decrease of the bandgap of PbGa_2_S_4_ under compression is due to two effects: the delocalized character of Pb 6s states and the higher compressibility of dodecahedral units, PbS_8_, compared to tetrahedral units, GaS_4_. The same behavior has been previously observed in other lead compounds, such as PbWO_4_, PbMoO_4_, and PbCrO_4_.^42,70–72^

In Fig. 10 we have also shown that the indirect optical bandgap of Pb_6_Ga_10_S_21_ also decreases under compression. This behavior is also consistent with the theoretical pressure dependence of the indirect optical bandgap of this compound from our metaGGA calculations as plotted in Fig. 10. Our calculations show that Pb_6_Ga_10_S_21_ is also a semiconductor with an indirect bandgap of 0.74 eV followed by a direct bandgap at 0.95 eV at 18.2 GPa. This means that our calculations underestimate the value of the indirect bandgap by 0.8 eV. The mechanism exposed in the previous paragraph to explain the decrease of the bandgaps of PbGa_2_S_4_ with pressure is also valid to explain the decrease of the bandgaps of Pb_6_Ga_10_S_21_, as evidenced by the similarity between the theoretical electronic band structure and electronic density of states of Pb_6_Ga_10_S_21_ at 15.3 GPa (shown in Fig. S7 in the ESI†) and those already described for PbGa_2_S_4_.

## 4 Conclusions

We have reported the effect of pressure on the structural, vibrational, and optical properties of lead thiogallate by means of powder HP-XRD, HP-RS, and HP-OA measurements beyond 20 GPa. Those measurements have been complemented with *ab initio* calculations at HP. First of all, we have checked that PbGa_2_S_4_ crystallizes at room conditions in the EuGa_2_S_4_-type orthorhombic (space group *Fddd*) structure by means of single-crystal XRD measurements. Then, we have shown by means of powder HP-XRD measurements that PbGa_2_S_4_ is an anisotropic material, as expected from its layered-like structure. Its axial and bulk moduli are of the same order as those of monoclinic α′-Ga_2_S_3_ and tetragonal CdGa_2_S_4_ and HgGa_2_S_4_; *i.e.* semiconductors with similar GaS_4_ tetrahedra.

After checking the complex vibrational pattern of PbGa_2_S_4_ by means of polarized RS measurements at room conditions, we have shown that the vibrational modes of PbGa_2_S_4_ show phonon spectra that show a considerable similarity to those of α′-Ga_2_S_3_, CdGa_2_S_4_, and HgGa_2_S_4_. In fact, we have shown that the pressure dependence of the Raman-active modes in PbGa_2_S_4_ is similar to those of the mentioned semiconductors and have made a tentative assignment of the symmetry of the Raman-active modes experimentally observed.

Finally, we have measured the pressure dependence of the optical bandgap of PbGa_2_S_4_ by means of HP-OA measurements. We have confirmed that PbGa_2_S_4_ is an indirect bandgap semiconductor, whose bandgap decreases as pressure increases, unlike what happens in α′-Ga_2_S_3_, CdGa_2_S_4_, and HgGa_2_S_4_. The different behavior is explained by the contribution of the 6s lone electron pair of Pb to the topmost valence band and the strong decrease of the Pb–S bond distance upon compression that leads to a strong increase of the energy of the topmost valence band under pressure.

To finish, we want to stress that all our measurements have shown evidence of a partially reversible pressure-induced decomposition of PbGa_2_S_4_ into a mixture of Pb_6_Ga_10_S_21_ and β′-Ga_2_S_3_ above 16 GPa. This decomposition is supported by enthalpy *vs.* pressure calculations of the three compounds and makes sense because both compounds show sixfold-coordinated Ga atoms in comparison with the fourfold-coordinated Ga atoms in PbGa_2_S_4_. The structure of the new compound Pb_6_Ga_10_S_21_ at HP, which is isostructural to already known Pb_6_In_10_S_21_ at RP, is reported at 23.5 GPa since it seems not to be stable at RP. Moreover, we have determined its Raman-active phonons and optical bandgap above 16 GPa. In summary, this work shows the first HP study of a compound with EuGa_2_S_4_-type (orthorhombic *Fddd*) structure and the route for the synthesis of Pb_6_Ga_10_S_21_. Therefore, this work will be of interest for the study of the EuGa_2_S_4_-type subfamily of A^II^B^III^_2_X^VI^_4_ compounds, in which the effect of pressure is far from being understood.

## Author contributions

Tania Garcia-Sanchez: investigation, formal analysis, discussion, writing, review and editing Samuel Gallego-Parra: investigation, formal analysis Akun Liang: investigation, formal analysis José Luis Rodrigo-Ramon: investigation, formal analysis Alfonso Muñoz: investigation, formal analysis Plácida Rodriguez-Hernandez: investigation, formal analysis Javier Gonzalez-Platas: investigation, formal analysis Juan Ángel Sans: investigation Vanesa Paula Cuenca-Gotor: investigation Hussien H. Osman: investigation Catalin Popescu: investigation Veaceslav Ursaki: investigation Ion M. Tiginyanu: investigation Daniel Errandonea: formal analysis, validation, funding acquisition, writing, review, and editing Francisco Javier Manjón: conceptualization, investigation, formal analysis, validation, writing, review, and editing, funding acquisition.

## Conflicts of interest

There are no conflicts to declare.

## Supplementary Material

TC-011-D3TC02288A-s001

## References

[cit1] Georgobiani A. N., Tagiev B. G., Tagiev O. B., Djabbarov R. B., Musaeva N. N., Kasumov U. F. (2000). Jpn. J. Appl. Phys..

[cit2] Peters T., Baglio J. (1972). J. Electrochem. Soc..

[cit3] Kaminskii A. A. (2007). Laser Photonics Rev..

[cit4] Shiryaev V., Churbanov M. (2017). J. Non-Cryst. Solids.

[cit5] Mirov S. B., Moskalev I. S., Vasilyev S., Smolski V., Fedorov V. V., Martyshkin D., Peppers J., Mirov M., Dergachev A., Gapontsev V. (2018). IEEE J. Sel. Top. Quantum Electron..

[cit6] Basiev T., Doroshenko M., Osiko V., Badikov D. (2005). Adv. photonics.

[cit7] Doroshenko M. E., Basiev T. T., Osiko V. V., Badikov V. V., Badikov D. V., Jelnková H., Koranda P., Šulc J. (2009). Opt. Lett..

[cit8] Šulc J., Jelnková H., Doroshenko M. E., Basiev T. T., Osiko V. V., Badikov V. V., Badikov D. V. (2010). Opt. Lett..

[cit9] Jelnková H., Doroshenko M., Jelnek M., Šulc J., Basiev T., Osiko V., Badikov V., Badikov D. (2011). Laser Phys. Lett..

[cit10] Jelnková H., Doroshenko M. E., Jelnek M., Šulc J., Osiko V. V., Badikov V. V., Badikov D. V. (2013). Opt. Lett..

[cit11] Yu X., Huang C., Ni Y., Wang Z., Wu H. (2022). CrystEngComm.

[cit12] Huang C., Ni Y., Wu H., Wang Z., Jiang P., Han W. (2020). Cryst. Growth Des..

[cit13] Chilouet A., Mazurier A., Guittard M. (1979). Mater. Res. Bull..

[cit14] Wu K., Pan S., Wu H., Yang Z. (2015). J. Mol. Struct..

[cit15] Bletskan D., Voroshilov Y. V., Durdinets L., Kabacij V. (1999). Sci. Her. Uzhhorod Univ. Ser. Phys..

[cit16] Shannon R. D. (1976). Acta Crystallogr., Sect. A: Cryst. Phys., Diffr., Theor. Gen. Crystallogr..

[cit17] Syrbu N., Lvin V., Zadnipui B., Golovei M., Poluprov F. T. (1991). Phys. Tech. Semicond.

[cit18] Neumann H., Sobotta H., Syrbu N., Golovei V. (1994). Cryst. Res. Technol..

[cit19] Syrbu N., Cebotari V. (1998). J. Phys.: Condens. Matter.

[cit20] Zalamai V., Syrbu N., Bejan N., Hirjeu I. (2016). Mater. Sci..

[cit21] Kamenshchikov V., Stefanovich V., Gadmashi Z., Side V., Suslikov L. (2007). Phys. Solid State.

[cit22] Golovey V., Ivanchenko L., Knyazev A., Obolonchik V., Troyan E. (1981). Ukr. fiz. ž..

[cit23] Neumann H., Hörig W., Nooke G., Syrbu N. (1988). Solid State Commun..

[cit24] Badikov V., Badikov D., Doroshenko M., Panyutin V., Chizhikov V., Shevyrdyaeva G. (2008). Opt. Mater..

[cit25] Syrbu N., Parvan V., Ursaki V. (2012). Opt. Mater..

[cit26] Stamov I., Syrbu N., Ursaki V., Parvan V., Zalamai V. (2012). Opt. Commun..

[cit27] Chen W.-F., Liu B.-W., Jiang X.-M., Guo G.-C. (2022). J. Alloys Compd..

[cit28] Syrbu N. and Zalamai V.

[cit29] Musaeva N., Dzhabbarov R., Kasumov U., Ganbarova K. B. (2003). J. Opt. Technol..

[cit30] Manjon F. J. , Tiginyanu I. and Ursaki V., Pressure-induced phase transitions in AB2X4 chalcogenide compounds, Springer, 2014

[cit31] Isaenko L., Yelisseyev A., Tkachuk A., Ivanova S. (2008). NATO Science for Peace and Security Series B: Physics and Biophysics.

[cit32] Orlovskii Y. V., Basiev T. T., Pukhov K. K., Doroshenko M. E., Badikov V. V., Badikov D. V., Alimov O. K., Polyachenkova M. V., Dmitruk L. N., Osiko V. V. (2007). et al.. Opt. Mater..

[cit33] Errandonea D. (2017). Phys. Status Solidi B.

[cit34] Mao H., Xu J.-A., Bell P. (1986). J. Geophys. Res. Solid Earth.

[cit35] Dewaele A., Loubeyre P., Mezouar M. (2004). Phys. Rev. B: Condens. Matter Mater. Phys..

[cit36] Fauth F., Peral I., Popescu C., Knapp M. (2013). Powder Diffr..

[cit37] Prescher C., Prakapenka V. B. (2015). High Press. Res..

[cit38] Toby B. H., Von Dreele R. B. (2013). J. Appl. Crystallogr..

[cit39] Letoullec R., Pinceaux J., Loubeyre P. (1988). High Press. Res..

[cit40] Gomis O., Vilaplana R., Manjón F. J., Ruiz-Fuertes J., Pérez-González E., López-Solano J., Bandiello E., Errandonea D., Segura A., Rodrguez-Hernández P. (2015). et al.. Phys. Status Solidi B.

[cit41] Errandonea D., Popescu C., Garg A. B., Botella P., Martinez-García D., Pellicer-Porres J., Rodríguez-Hernández P., Muñoz A., Cuenca-Gotor V., Sans J. A. (2016). Phys. Rev. B: Condens. Matter Mater. Phys..

[cit42] Errandonea D., Martinez-Garcia D., Lacomba-Perales R., Ruiz-Fuertes J., Segura A. (2006). Appl. Phys. Lett..

[cit43] Hohenberg P., Kohn W. (1964). Phys. Rev..

[cit44] Kresse G., Hafner J. (1993). Phys. Rev. B: Condens. Matter Mater. Phys..

[cit45] Perdew J. P., Burke K., Ernzerhof M. (1996). Phys. Rev. Lett..

[cit46] Mujica A., Rubio A., Munoz A., Needs R. (2003). Rev. Mod. Phys..

[cit47] Tao J., Perdew J. P., Staroverov V. N., Scuseria G. E. (2003). Phys. Rev. Lett..

[cit48] Becke A. D., Johnson E. R. (2006). J. Chem. Phys..

[cit49] Errandonea D., Kumar R. S., Gomis O., Manjón F. J., Ursaki V. V., Tiginyanu I. M. (2013). J. Appl. Phys..

[cit50] Krämer V., Berroth K. (1980). Mater. Res. Bull..

[cit51] Gallego-Parra S., Vilaplana R., Gomis O., da Silva E. L., Otero-de-la Roza A., Rodrguez-Hernández P., Muñoz A., González J., Sans J., Cuenca-Gotor V. P. (2021). et al.. Phys. Chem. Chem. Phys..

[cit52] Gallego-Parra S., Vilaplana R., Gomis O., Rodríguez-Hernández P., Munoz A., González J. A., Sans J. A., Popescu C., Manjón F. J. (2022). Chem. Mater..

[cit53] Jiang Q., Li R., Wang F., Shi X., Chen F., Huang Y., Wang B., Zhang W., Wu X., Wei F. (2022). et al.. Adv. Mater..

[cit54] Dunuwille M., Kim M., Yoo C.-S. (2016). J. Chem. Phys..

[cit55] Errandonea D., Kumar R. S., Manjón F. J., Ursaki V., Tiginyanu I. (2008). J. Appl. Phys..

[cit56] Gomis O., Santamaria-Perez D., Vilaplana R., Luna R., Sans J., Manjón F. J., Errandonea D., Perez-Gonzalez E., Rodrguez-Hernández P., Muñoz A. (2014). et al.. J. Alloys Compd..

[cit57] Birch F. (1947). Phys. Rev..

[cit58] Angel R. J., Alvaro M., Gonzalez-Platas J. (2014). Z. Kristallogr. - Cryst. Mater..

[cit59] Angel R., Mosenfelder J., Shaw C. (2001). Phys. Earth Planet. Inter..

[cit60] Gomis O., Vilaplana R., Manjón F. J., Santamaria-Perez D., Errandonea D., Perez-Gonzalez E., Lopez-Solano J., Rodriguez-Hernandez P., Munoz A., Tiginyanu I. M. (2013). et al.. Mater. Res. Bull..

[cit61] Errandonea D., Muñoz A., Gonzalez-Platas J. (2014). J. Appl. Phys..

[cit62] Gallego-Parra S., Gomis O., Vilaplana R., Ortiz H. M., Perez-Gonzalez E., Luna R., Rodrguez-Hernández P., Muñoz A., Ursaki V., Tiginyanu I. (2019). et al.. J. Appl. Phys..

[cit63] Fuentes-Cabrera M. (2001). J. Phys.: Condens. Matter.

[cit64] Kroumova E., Aroyo M., Perez-Mato J., Kirov A., Capillas C., Ivantchev S., Wondratschek H. (2003). Phase Transitions.

[cit65] Vilaplana R., Robledillo M., Gomis O., Sans J., Manjón F. J., Perez-Gonzalez E., Rodrguez-Hernández P., Muñoz A., Tiginyanu I., Ursaki V. (2013). J. Appl. Phys..

[cit66] Garg A. B., Vie D., Rodriguez-Hernandez P., Muñoz A., Segura A., Errandonea D. (2023). J. Phys. Chem. Lett..

[cit67] Liang A., Rodríguez F., Rodríguez-Hernandez P., Muñoz A., Turnbull R., Errandonea D. (2022). Phys. Rev. B: Condens. Matter Mater. Phys..

[cit68] Liang A., Shi L., Gallego-Parra S., Gomis O., Errandonea D., Tiginyanu I., Ursaki V., Manjón F. (2021). J. Alloys Compd..

[cit69] Manjón F. J., Gomis O., Rodrguez-Hernández P., Perez-Gonzalez E., Muñoz A., Errandonea D., Ruiz-Fuertes J., Segura A., Fuentes-Cabrera M., Tiginyanu I. (2010). et al.. Phys. Rev. B: Condens. Matter Mater. Phys..

[cit70] Errandonea D., Bandiello E., Segura A., Hamlin J., Maple M., Rodriguez-Hernandez P., Muñoz A. (2014). J. Alloys Compd..

[cit71] Monteseguro V., Ruiz-Fuertes J., Contreras-Garca J., Rodrguez-Hernández P., Muñoz A., Errandonea D. (2019). Appl. Phys. Lett..

[cit72] Bandiello E., Errandonea D., Martinez-Garcia D., Santamaria-Perez D., Manjón F. J. (2012). Phys. Rev. B: Condens. Matter Mater. Phys..

