# Supplementary material for: Joint experimental and theoretical study of PbGa2S4 under compression
Source: J Mater Chem C Mater. 2023 Jul 28;11(34):11606–19. doi: 10.1039/d3tc02288a (PMC10469446; doi:10.1039/d3tc02288a)
Supplement: TC-011-D3TC02288A-s001 [file TC-011-D3TC02288A-s001.pdf]

Electronic Supplementary Material (ESI) for Journal of Materials Chemistry C.
This journal is © The Royal Society of Chemistry 2023

Supporting Information of Joint experimental and theoretical study of $PbGa_2S_4$ under compression

T. Garcia-Sanchez[a], S. Gallego-Parra[b,*], A. Liang[c,**], J.L Rodrigo- Ramon[c], A. Muñoz[d], P. Rodriguez-Hernandez[d], J. Gonzalez-Platas[d], J.A Sans[b], V.P Cuenca-Gotor[b], Hussien H. Osman, C[c]. Popescu[e], Veaceslav Ursaki[f], Ion M. Tiginyanu[f], D. Errandonea[c] and F.J. Manjón[b]

[a]*Departamento de Ingeniería Eléctica, MALTA Consolider Team, Universitat Politècnica de València, Camino de Vera, s/n., Valencia, 46022, Spain*

[b]*Instituto de Diseño para la Fabricación y Producción Automatizada, MALTA Consolider Team, Universitat Politècnica de València, Camino de Vera, s/n., Valencia, 46022, Spain*

[c]*Departamento de Física Aplicada-ICMUV, MALTA Consolider Team, Universitat de Valencia, Dr. Moliner 50, Burjassot, Valencia, 46100, Spain,*

[d]*Departamento de Física, Instituto de Materiales y Nanotecnología, MALTA Consolider Team, Universidad de La Laguna, La Laguna, Tenerife, 38205, Spain*

[e]*ALBA-CELLS MALTA Consolider Team Cerdanyola del Valles (Barcelona) Cataluña 08290 Spain*

[f]*National Center for Materials Study and Testing, Technical University of Moldova, Chisinau MD-2004, Republic of Moldova*

Present address: [*], European Synchrotron Radiation Facility, 71 Avenue des Martyrs, 38000 Grenoble, France, and [**]. Centre for Science at Extreme Conditions and School of Physics and Astronomy, University of Edinburgh, Edinburgh EH9 3FD, United Kingdom.

1.- Single crystal XRD measurements at room pressure

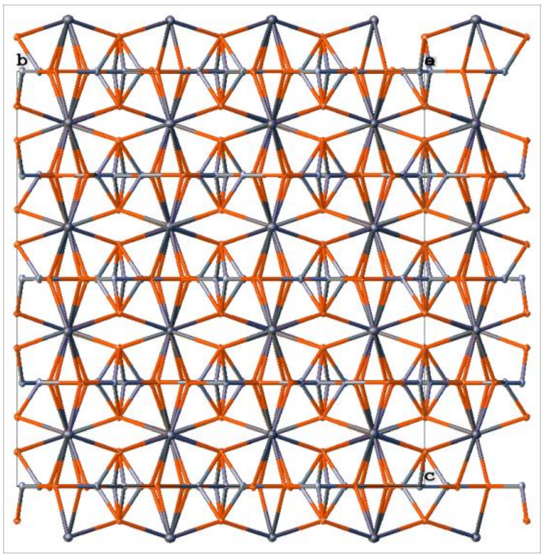

Figure S1. Detail of the crystal structure of $PbGa_2S_4$ along the *b* and *c* axes. Pb, Ga, and S atoms are big gray, small gray, and orange circles, respectively.

Experimental. Single orange plate crystals of $PbGa_2S_4$ were used. A suitable crystal with dimensions 0.12 × 0.11 × 0.04 mm^3 was selected and mounted on a SuperNova, Dualflex, EosS2 diffractometer. The crystal was kept at a steady T = 293(2) K during data collection. The diffraction data was collected with the CrysAlis Pro software **[S1]** and the structure was solved with the ShelXT 2018/2 **[S2,S3]** solution program using dual methods and by using Olex2 **[S4]** as the graphical interface. The model was refined with ShelXL 2018/3 using full matrix least squares minimisation on $\boldsymbol{F}^2$.

The structure was solved and the space group *Fddd* (# 70) determined by the ShelXT 2018/2 **[S2,S3]** structure solution program using using dual methods and refined by full matrix least squares minimisation on $\boldsymbol{F}^2$ using version 2018/3 of ShelXL 2018/3 **[S2,S3]**. All non-hydrogen atoms were refined anisotropically. Sulfur atom positions were calculated geometrically and refined using the riding model.

**Compound**	$\mathbf{PbGa_2S_4}$
Formula	$PbGa_2S_4$
$D_{calc.}$/ g cm^{-3}	4.907
μ/mm^{-1}	35.610
Formula Weight	474.87
Colour	orange
Shape	plate
Size/mm^3	0.12×0.11×0.04
T/K	293(2)
Crystal System	orthorhombic
Space Group	*Fddd*
a/Å	12.1674(2)
b/Å	20.4180(4)
c/Å	20.7005(5)
α/°	90
β/°	90
γ/°	90
V/Å^3	5142.71(18)
Z	32
Z'	1
Wavelength/Å	0.71073
Radiation type	Mo K_α
Θ_{min}/°	2.183
Θ_{max}/°	28.278
Measured Refl's.	24555
Indep't Refl's	1597
Refl's I≥2 σ(I)	1195
R_{int}	0.0353
Parameters	66
Restraints	0
Largest Peak	1.129
Deepest Hole	-0.841
GooF	1.185
wR_2 (all data)	0.0520
wR_2	0.0480
R_1 (all data)	0.0357
R_1	0.0236

Table S1: Anisotropic Displacement Parameters (×10^4) for $PbGa_2S_4$. The anisotropic displacement factor exponent takes the form: $-2\pi^2[h^2a^{*2} \times U_{11} + ... + 2hka^* \times b^* \times U_{12}]$

**Atom**	$\boldsymbol{U_{11}}$	$\boldsymbol{U_{22}}$	$\boldsymbol{U_{33}}$	$\boldsymbol{U_{23}}$	$\boldsymbol{U_{13}}$	$\boldsymbol{U_{12}}$
Pb3	20.4(2)	23.2(2)	22.7(3)	0	0	0
Pb2	15.20(18)	24.9(2)	24.6(2)	0	0	3.29(10)
Pb1	15.9(2)	33.8(3)	22.9(3)	0	0	0
Ga1	7.3(2)	11.6(2)	18.8(3)	0.0(2)	0.1(2)	-0.98(18)
Ga2	7.4(2)	12.1(3)	19.1(3)	-0.1(2)	0.3(2)	-0.64(17)
S2	10.5(5)	12.4(5)	14.7(5)	2.4(8)	-2.4(8)	-0.1(4)
S3	9.5(4)	14.6(5)	14.4(5)	-2.2(8)	2.3(8)	1.9(4)

Atom	U_{11}	U_{22}	U_{33}	U_{23}	U_{13}	U_{12}
S4	5.7(4)	10.6(5)	18.3(5)	-1.4(6)	0.5(6)	-0.1(3)
S1	7.7(4)	10.0(5)	21.3(6)	-0.4(6)	1.4(6)	-1.5(4)

Table S2: Bond Lengths in Å for $PbGa_2S_4$.

Atom	Atom	Length/Å
Pb3	S3^{1}	3.0918(19)
Pb3	S3^{2}	3.0918(19)
Pb3	S3^{3}	3.0918(19)
Pb3	S3^{4}	3.0918(19)
Pb3	S4^{5}	3.0801(19)
Pb3	S4^{6}	3.0801(19)
Pb3	S4^{7}	3.0801(19)
Pb3	S4	3.0802(19)
Pb2	S2^{8}	3.0772(18)
Pb2	S2^{9}	3.0772(18)
Pb2	S3^{10}	3.1117(18)
Pb2	S3^{11}	3.1117(18)
Pb2	S4^{10}	3.0769(19)
Pb2	S4^{11}	3.0769(19)
Pb2	S1^{12}	3.059(2)
Pb2	S1	3.059(2)
Pb1	S2^{6}	3.0973(19)
Pb1	S2^{13}	3.0973(19)
Pb1	S2	3.0974(19)
Pb1	S2^{12}	3.0973(19)
Pb1	S1^{12}	3.076(2)
Pb1	S1^{6}	3.076(2)
Pb1	S1	3.076(2)
Pb1	S1^{13}	3.0762(19)
Ga1	S2^{8}	2.3026(16)
Ga1	S2	2.2952(16)
Ga1	S4	2.2443(11)
Ga1	S1	2.3010(12)
Ga2	S3	2.2987(16)
Ga2	S3^{4}	2.3031(16)
Ga2	S4^{9}	2.3023(12)
Ga2	S1	2.2451(12)

----
^{1}1/4+x,1-y,1/4+z; ^{2}3/2-x,-1/4+y,1/4+z; ^{3}1/4+x,-1/4+y,1/2-z; ^{4}3/2-x,1-y,1/2-z; ^{5}7/4-x,+y,3/4-z; ^{6}+x,3/4-y,3/4-z; ^{7}7/4-x,3/4-y,+z; ^{8}1-x,1/2-y,1/2-z; ^{9}-1/4+x,1/4+y,1/2-z; ^{10}5/4-x,+y,1/4-z; ^{11}-1/2+x,3/4-y,1/4-z; ^{12}3/4-x,3/4-y,+z; ^{13}3/4-x,+y,3/4-z

Table S3: Bond Angles (in °) for $PbGa_2S_4$.

Atom	Atom	Atom	Angle
S3^{1}	Pb3	S3^{2}	147.38(4)
S3^{3}	Pb3	S3^{4}	147.38(4)
S3^{1}	Pb3	S3^{4}	68.51(7)
S3^{2}	Pb3	S3^{3}	68.51(7)
S3^{1}	Pb3	S3^{3}	121.60(7)
S3^{2}	Pb3	S3^{4}	121.60(7)
S4^{5}	Pb3	S3^{2}	73.89(3)
S4	Pb3	S3^{3}	73.89(3)
S4^{5}	Pb3	S3^{3}	77.00(3)
S4^{6}	Pb3	S3^{4}	77.00(3)
S4^{5}	Pb3	S3^{1}	78.54(3)
S4^{7}	Pb3	S3^{3}	78.54(3)
S4^{6}	Pb3	S3^{1}	73.89(3)
S4^{7}	Pb3	S3^{2}	134.53(3)
S4	Pb3	S3^{4}	78.54(3)
S4^{5}	Pb3	S3^{4}	134.53(3)
S4	Pb3	S3^{1}	134.53(3)
S4^{6}	Pb3	S3^{2}	78.54(3)
S4^{6}	Pb3	S3^{3}	134.53(3)
S4	Pb3	S3^{2}	77.00(3)
S4^{7}	Pb3	S3^{4}	73.89(3)
S4^{7}	Pb3	S3^{1}	77.00(3)
S4^{5}	Pb3	S4	144.60(5)
S4^{7}	Pb3	S4	64.02(5)
S4^{6}	Pb3	S4	128.53(5)
S4^{5}	Pb3	S4^{7}	128.52(5)
S4^{5}	Pb3	S4^{6}	64.02(5)
S4^{6}	Pb3	S4^{7}	144.60(5)
S2^{8}	Pb2	S2^{9}	149.22(5)
S2^{9}	Pb2	S3^{10}	68.90(6)
S2^{8}	Pb2	S3^{10}	121.04(6)
S2^{9}	Pb2	S3^{11}	121.04(6)
S2^{8}	Pb2	S3^{11}	68.90(6)
S3^{10}	Pb2	S3^{11}	145.88(5)
S4^{10}	Pb2	S2^{9}	133.70(3)
S4^{11}	Pb2	S2^{9}	76.05(3)
S4^{10}	Pb2	S2^{8}	76.05(3)
S4^{11}	Pb2	S2^{8}	133.70(3)
S4^{10}	Pb2	S3^{10}	77.67(3)
S4^{11}	Pb2	S3^{10}	73.52(3)
S4^{10}	Pb2	S3^{11}	73.52(3)
S4^{11}	Pb2	S3^{11}	77.67(3)
S4^{10}	Pb2	S4^{11}	64.10(5)
S1^{12}	Pb2	S2^{9}	81.89(4)
S1^{12}	Pb2	S2^{8}	72.40(3)
S1	Pb2	S2^{8}	81.89(4)
S1	Pb2	S2^{9}	72.41(3)
S1^{12}	Pb2	S3^{10}	79.97(4)
S1	Pb2	S3^{11}	79.97(4)
S1	Pb2	S3^{10}	131.63(4)
S1^{12}	Pb2	S3^{11}	131.62(4)
S1	Pb2	S4^{10}	150.17(3)
S1	Pb2	S4^{11}	123.26(4)
S1^{12}	Pb2	S4^{10}	123.26(4)
S1^{12}	Pb2	S4^{11}	150.17(3)
S1^{12}	Pb2	S1	66.72(6)
S2^{13}	Pb1	S2	69.11(7)
S2^{6}	Pb1	S2	120.74(7)
S2^{12}	Pb1	S2	147.72(4)
S2^{6}	Pb1	S2^{12}	69.11(7)
S2^{6}	Pb1	S2^{13}	147.71(4)
S2^{13}	Pb1	S2^{12}	120.74(7)

Atom	Atom	Atom	Angle
S1[6]	Pb1	S2[6]	71.58(3)
S1	Pb1	S2[6]	79.29(3)
S1	Pb1	S2	71.58(3)
S1[12]	Pb1	S2[12]	71.58(3)
S1[12]	Pb1	S2[13]	79.29(3)
S1[13]	Pb1	S2[12]	79.29(3)
S1[6]	Pb1	S2[13]	81.40(3)
S1[13]	Pb1	S2[6]	81.40(3)
S1[12]	Pb1	S2	81.40(3)
S1[6]	Pb1	S2[12]	130.64(4)
S1[13]	Pb1	S2	130.64(4)
S1	Pb1	S2[13]	130.64(4)
S1[13]	Pb1	S2[13]	71.58(3)
S1[6]	Pb1	S2	79.29(3)
S1	Pb1	S2[12]	81.40(3)
S1[12]	Pb1	S2[6]	130.64(4)
S1[6]	Pb1	S1[13]	66.30(6)
S1[13]	Pb1	S1	156.51(4)
S1[6]	Pb1	S1	119.00(5)
S1[12]	Pb1	S1[6]	156.51(4)
S1[12]	Pb1	S1[13]	119.00(5)
S1[12]	Pb1	S1	66.30(6)
S2	Ga1	S2[9]	97.35(4)
S2	Ga1	S1	103.54(7)
S4	Ga1	S2[9]	123.09(7)
S4	Ga1	S2	123.61(7)
S4	Ga1	S1	102.62(4)
S1	Ga1	S2[9]	103.87(7)
S3	Ga2	S3[4]	96.70(4)
S3	Ga2	S4[8]	107.46(7)
S4[8]	Ga2	S3[4]	107.06(7)
S1	Ga2	S3	120.92(7)
S1	Ga2	S3[4]	121.33(7)
S1	Ga2	S4[8]	102.42(4)
Pb2[9]	S2	Pb1	148.47(4)
Ga1	S2	Pb2[9]	112.28(7)
Ga1[9]	S2	Pb2[9]	91.35(6)
Ga1	S2	Pb1	91.99(6)
Ga1[9]	S2	Pb1	112.26(7)
Ga1	S2	Ga1[9]	82.65(4)
Pb3[4]	S3	Pb2[11]	146.63(4)
Ga2	S3	Pb3[4]	88.97(6)
Ga2[4]	S3	Pb3[4]	116.12(7)
Ga2	S3	Pb2[11]	116.97(7)
Ga2[4]	S3	Pb2[11]	88.98(6)
Ga2	S3	Ga2[4]	83.30(4)
Pb2[11]	S4	Pb3	115.94(3)
Ga1	S4	Pb3	118.92(8)
Ga1	S4	Pb2[11]	120.06(8)
Ga1	S4	Ga2[3]	112.76(5)
Ga2[3]	S4	Pb3	89.19(5)
Ga2[3]	S4	Pb2[11]	89.85(5)
Pb2	S1	Pb1	113.49(3)
Ga1	S1	Pb2	91.84(6)
Ga1	S1	Pb1	92.42(6)
Ga2	S1	Pb2	119.41(8)
Ga2	S1	Pb1	119.40(8)
Ga2	S1	Ga1	112.57(5)

----

[1]1/4+x,1-y,1/4+z; [2]3/2-x,-1/4+y,1/4+z; [3]1/4+x,-1/4+y,1/2-z; [4]3/2-x,1-y,1/2-z; [5]7/4-x,+y,3/4-z; [6]+x,3/4-y,3/4-z; [7]7/4-x,3/4-y,+z; [8]-1/4+x,1/4+y,1/2-z; [9]1-x,1/2-y,1/2-z; [10]-1/2+x,3/4-y,1/4-z; [11]5/4-x,+y,1/4-z; [12]3/4-x,3/4-y,+z; [13]3/4-x,+y,3/4-z

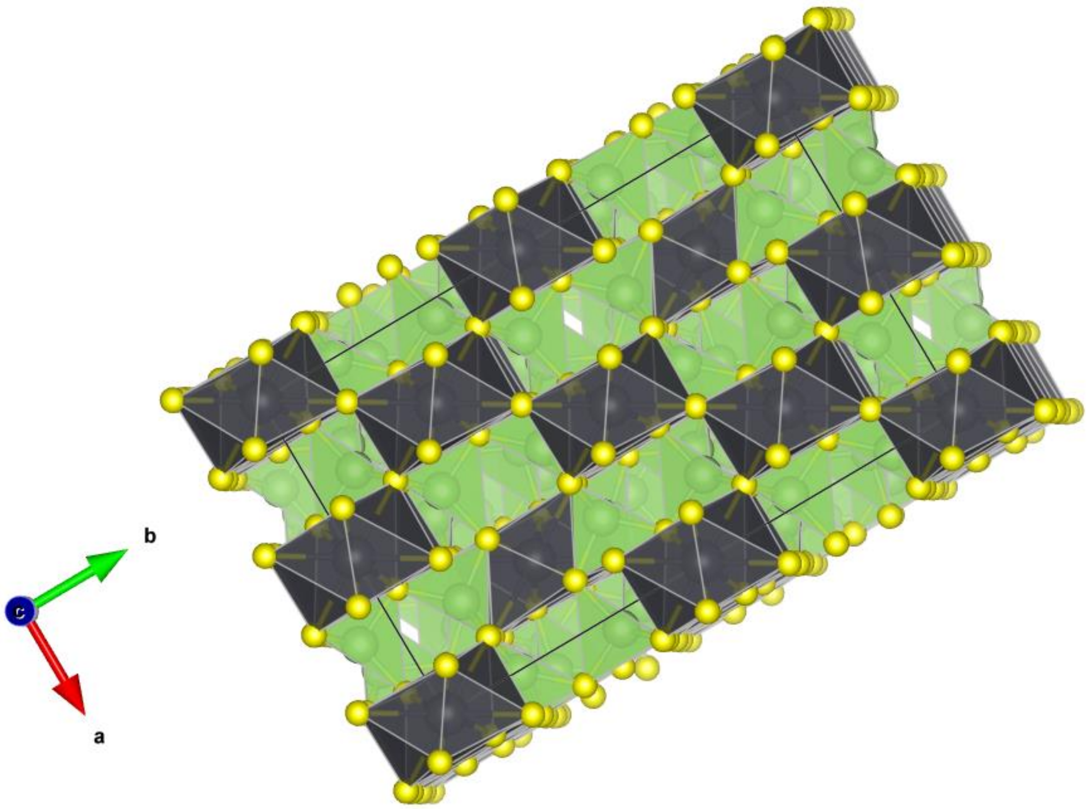

Figure S2. Detail of the crystal structure of $PbGa_2S_4$ in a perspective that highlights its layered characteristic. GaS_4 tetrahedra are shown in green and PbS_8 square antiprismatic polyhedra is black. Sulfur atoms are shown in yellow.

2.- RS measurements at room pressure

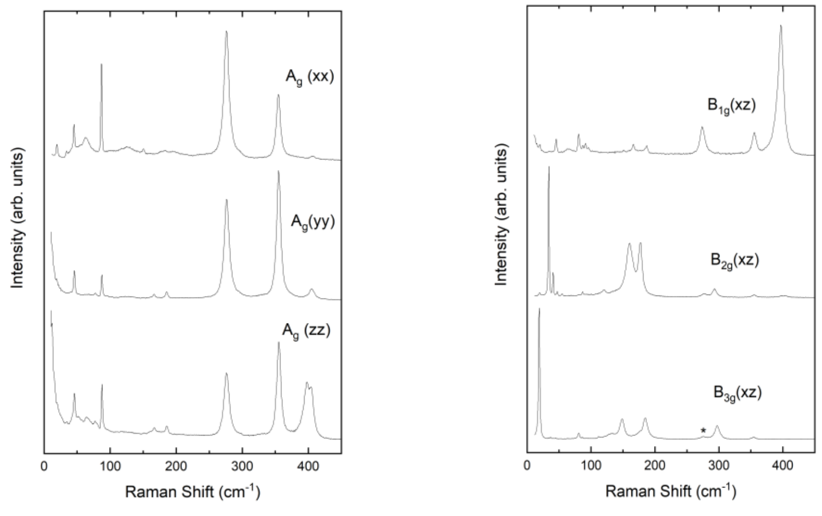

(a) (b)

Figure S3. Normalized Raman spectra of $PbGa_2S_4$ at room pressure. (a) Modes with A_g symmetry. (b) Modes with B_g symmetry. The asterisk in the Raman spectrum corresponding to the B_{3g} symmetry is related to an A_g (xx) mode.

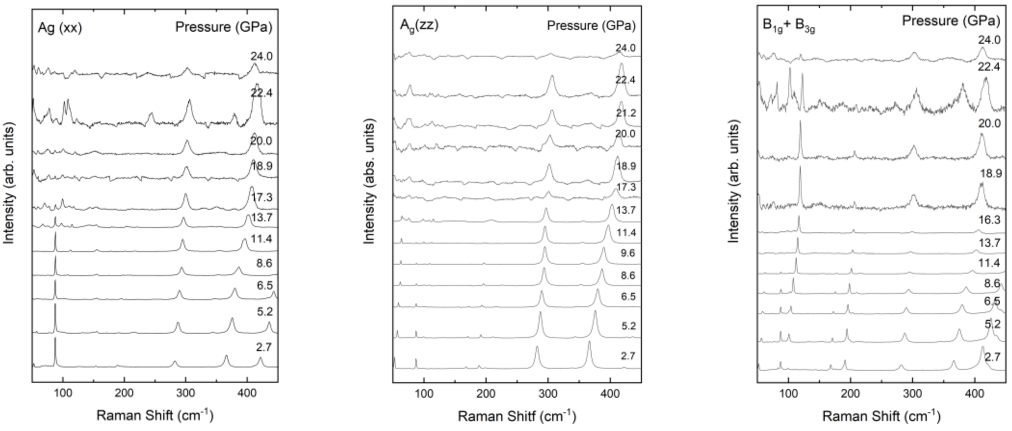

Figure S4. Normalized polarized Raman spectra of $PbGa_2S_4$ at high pressure up to 24 GPa.

Table S4

Raman-active frequencies of A_g, B_{1g}, B_{2g} and B_{3g} modes in $PbGa_2S_4$ at room pressure obtained from polarized RS measurements. Data from Ref. Kamenshchikov et al. (2007) (noted with subindex k) are provided for comparison.

A_{gk}	A_g	B_{1gk}	B_{1g}	B_{2gk}	B_{2g}	B_{3gk}	B_{3g}
	12.2		20	19.7	20	19	
	19.7	25		34	33.9	31	34
	20.3	35	34.3	41	40.7		
	22.4	42		47	47.2	47	45
	25	46	45.6	55	55.0	55	
	34.1	67	65	89	86.5	89	81
	34.9	83	80.5	121	121		112
	45.5		87	160	160.8	132	131
46	46.2	93	91.2	176	177.0	150	149
53	51.9	98	96.5		277.0	175	179
	62.7	152		293	293.1	187	184
67	64.0	161	166.2	362	354.8	280	277
80	77.0	167			398.6	298	297
	86.8	178				336	330
88	87.6	187	187.2			345	355
118		274	273.7				
127	125.7	292					
159	150.8	321					
166	166.6	356	355				
172		398	396.9				
	182.0						
185	185.4						
	198.5						
277	276.4						
332							
353	355.0						
	397.8						
404	404.1						

Table S5
Theoretical Raman-active frequencies at zero pressure (ω_0, in cm^{-1}) and pressure coefficients (a_1, in $cm^{-1}GPa^{-1}$; a_2, in $cm^{-1}GPa^{-2}$) in $PbGa_2S_4$ according to fits to ω_0+a_1·P+ a_2·P^2.

Mode	ω_0	a_1	a_2	Mode	ω_0	a_1	a_2
B_{2g}^1	17.7(2)	3.81(0)	-0.1(0)	B_{2g}^{12}	162.9(2)	3.3(0)	0.0(0)
B_{1g}^1	30.4(1)	2.2(0)	-0.1(0)	B_{2g}^{13}	167.1(1)	4.1(0)	0.0(0)
B_{3g}^1	33.8(1)	0.7(0)	-0.1(0)	B_{3g}^{13}	167.3(1)	4.4(0)	0.0(0)
B_{2g}^2	36.46(1)	1.8(0)	0.0(0)	B_{2g}^{14}	169.7(1)	4.3(0)	0.0(0)
B_{3g}^2	39.2(0)	1.6(0)	-0.1(0)	B_{3g}^{14}	173.9(0)	5.8(0)	0.0(0)
B_{1g}^2	43.6(1)	2.4(0)	0.0(0)	A_g^9	170.5(2)	3.2(0)	0.0(0)
A_g^1	44.0(1)	2.4(0)	-0.1(0)	B_{1g}^{12}	174.2(1)	3.3(0)	0.0(0)
B_{3g}^3	46.4(0)	1.8(0)	0.0(0)	B_{1g}^{13}	179.6(0)	5.3(0)	0.0(0)
B_{2g}^3	50.5(0)	1.8(0)	0.0(0)	A_g^{10}	182.3(0)	5.4(0)	0.0(0)
B_{3g}^4	53.8(0)	1.7(0)	0.0(0)	A_g^{11}	195.5(0)	4.8(0)	0.0(0)
B_{2g}^4	64.3(0)	2.7(0)	0.0(0)	B_{1g}^{14}	262.9(0)	2.2(0)	0.0(0)
B_{3g}^5	79.1(2)	1.8(0)	-0.1(0)	A_g^{12}	266.0(0)	2.0(0)	0.0(0)
B_{2g}^5	77.8(1)	2.1(0)	0.0(0)	B_{3g}^{15}	266.3(0)	3.4(0)	0.0(0)
B_{1g}^3	78.6(0)	0.6(0)	0.0(0)	B_{1g}^{15}	272.1(0)	3.7(0)	0.0(0)
B_{1g}^4	81.68(0)	0.7(0)	0.0(0)	B_{2g}^{15}	274.6(0)	3.7(0)	0.0(0)
A_g^2	84.3(0)	0.1(0)	0.0(0)	B_{1g}^{16}	276.0(0)	4.1(0)	0.0(0)
A_g^3	88.7(1)	1.1(0)	0.0(0)	B_{3g}^{16}	276.4(0)	4.3(0)	-0.1(0)
B_{1g}^5	88.8(1)	1.3(0)	0.0(0)	A_g^{13}	278.8(0)	4.0(0)	0.0(0)
B_{1g}^6	89.8(0)	1.9(0)	0.0(0)	B_{2g}^{16}	278.9(0)	4.5(0)	-0.1(0)
A_g^4	91.6(0)	1.1(0)	0.0(0)	A_g^{14}	283.3(0)	5.1(0)	-0.1(0)
B_{3g}^6	98.1(0)	-0.6(0)	0.0(0)	B_{2g}^{17}	292.0(0)	3.1(0)	0.0(0)
B_{3g}^7	108.3(0)	-0.4(0)	0.0(0)	B_{3g}^{17}	292.1(0)	3.2(0)	0.0(0)
B_{2g}^6	107.4(0)	-0.7(0)	0.0(0)	B_{3g}^{18}	292.7(0)	3.1(0)	0.0(0)
B_{2g}^7	110.4(0)	-0.7(0)	0.0(0)	B_{2g}^{18}	293.3(0)	3.0(0)	0.0(0)
B_{3g}^8	112.7(0)	0.0(0)	0.0(0)	A_g^{15}	320.0(1)	2.4(0)	0.0(0)
B_{2g}^8	113.7(0)	-0.1(0)	0.0(0)	B_{1g}^{17}	318.9(0)	3.0(0)	0.0(0)
B_{3g}^9	114.7(2)	7.7(0)	-0.2(0)	B_{3g}^{19}	334.4(0)	3.0(0)	0.0(0)
B_{2g}^9	128.8(3)	6.2(0)	-0.2(0)	B_{1g}^{18}	334.9(0)	3.5(0)	0.0(0)
B_{2g}^{10}	144.7(2)	4.7(0)	-0.1(0)	A_g^{16}	335.9(0)	3.5(0)	0.0(0)
B_{1g}^7	145.4(1)	0.8(0)	0.0(0)	A_g^{17}	336.9(0)	4.0(0)	0.0(0)
A_g^5	145.5(1)	0.1(0)	0.0(0)	B_{2g}^{19}	337.1(0)	3.0(0)	0.0(0)
A_g^6	148.7(1)	2.6(0)	0.0(0)	B_{1g}^{19}	338.1(0)	3.9(0)	0.0(0)

B_{1g}^{8}	151.5(1)	2.2(0)	0.0(0)	B_{3g}^{20}	338.1(0)	3.2(0)	0.0(0)
B_{3g}^{10}	153.5(2)	3.7(0)	-0.1(0)	B_{2g}^{20}	339.1(0)	3.2(0)	0.0(0)
A_{g}^{7}	149.7(1)	4.2(0)	-0.1(0)	B_{3g}^{21}	363.2(0)	5.0(0)	0.0(0)
B_{1g}^{9}	152.9(2)	4.7(0)	-0.1(0)	B_{2g}^{21}	369.4(0)	5.3(0)	-0.1(0)
B_{1g}^{10}	155.2(1)	5.1(1)	0.0(0)	B_{3g}^{22}	370.3(0)	5.3(0)	-0.1(0)
A_{g}^{8}	160.2(1)	3.2(2)	0.0(0)	B_{1g}^{20}	371.8(0)	5.8(0)	-0.1(0)
B_{3g}^{11}	166.4(2)	1.7(0)	0.0(0)	B_{2g}^{22}	374.7(0)	5.4(0)	-0.1(0)
B_{1g}^{11}	163.2(1)	4.3(0)	0.0(0)	A_{g}^{18}	379.2(0)	5.6(0)	-0.1(0)
B_{2g}^{11}	160.9(2)	2.8(0)	0.2(0)	A_{g}^{19}	380.0(0)	6.1(0)	-0.1(0)
B_{3g}^{12}	162.8(1)	4.7(0)	0.0(0)	B_{1g}^{21}	381.5(0)	5.3(0)	0.0(0)

Tabla S6

Theoretical Raman-active frequencies at 16 GPa (ω_{16}, in cm^{-1}) and pressure coefficients (a_1, in $cm^{-1}GPa^{-1}$; a_2, in $cm^{-1}GPa^{-2}$) in $Pb_6Ga_{10}S_{21}$ according to fits to ω_{16}+a_1·(P-16)+ a_2·(P-16)^2.

Mode	ω_0	a_1	a_2
Bg	43.5 (0)	2.0 (0)	-0.1 (0)
Bg	49.4 (0)	2.6 (0)	-0.1 (0)
Ag	48.0 (0)	0.4 (0)	0 (0)
Ag	54.4 (0)	0.9 (0)	0 (0)
Ag	56.4 (0	0.5 (0)	0 (0)
Bg	59.6 (0)	1.1 (0)	0 (0)
Bg	67.7 (0)	1.0 (0)	0 (0)
Ag	74.0 (0)	0.7 (0)	0 (0)
Ag	84.3 (0)	1.3 (0)	0 (0)
Bg	85.6 (0)	1.2 (0)	0 (0)
Bg	89.3 (0)	1.4 (0)	0 (0)
Ag	91.9 (0)	0.8 (0)	0 (0)
Ag	94.7 (0)	0.9 (0)	0 (0)
Bg	100.8 (0)	0.7 (0)	0 (0)
Ag	103.1 (0)	1.0 (0)	0 (0)
Ag	113.4 (0)	1.2 (0)	0 (0)
Bg	116.7 (0)	1.2 (0)	0 (0)
Ag	124.5 (0)	1.3 (0)	0 (0)
Ag	137.6 (0)	1.5 (0)	0 (0)
Ag	142.6 (0)	1.7 (0)	0 (0)
Bg	161.2 (0)	2.9 (0)	0 (0)
Ag	162.0 (0)	1.1 (0)	0 (0)
Ag	177.2 (0)	1.9 (0)	0 (0)
Ag	183.7 (0)	1.3 (0)	0 (0)
Ag	187.8 (0)	1.5 (0)	0 (0)
Ag	204.8 (0)	2.0 (0)	0 (0)
Bg	216.4 (0)	3.0 (0)	0 (0)
Ag	217,9 (0)	2.2 (0)	0 (0)
Ag	226.9 (0)	3.0 (0)	0 (0)
Bg	242,7 (0)	3.3 (0)	0 (0)
Ag	241.7 (0)	2.5 (0)	0 (0)
Bg	252.0 (0)	2.9 (0)	0 (0)
Bg	259.0 (0)	3.1 (0)	0 (0)
Ag	266.0 (0)	2.9 (0)	0 (0)
Bg	270.1 (0)	3.2 (0)	0 (0)
Ag	271.3 (0)	2.9 (0)	0 (0)
Ag	279.6 (0)	2.6 (0)	0 (0)
Bg	283.2 (0)	3.0 (0)	0 (0)
Ag	284.9 (0)	2.9 (0)	0 (0)
Bg	291.2 (0)	2.7 (0)	0 (0)
Ag	292.8 (0)	2.3 (0)	0 (0)
Bg	297.8 (0)	2.8 (0)	0 (0)

Bg	306.0 (0)	3.9 (0)	0 (0)
Ag	315.7 (0)	2.7 (0)	0 (0)
Ag	323.6 (0)	2.5 (0)	0 (0)
Ag	324.3 (0)	2.9 (0)	0 (0)
Ag	331.2 (0)	2.7 (0)	0 (0)
Ag	337.4 (0)	2.7 (0)	0 (0)
Ag	344.2 (0)	2.7 (0)	0 (0)
Ag	349.5 (0)	3.1 (0)	0 (0)
Ag	353.7 (0)	2.6 (0)	0 (0)
Ag	366.5 (0)	3.3 (0)	0 (0)
Ag	371.9 (0)	4.1 (0)	0 (0)
Ag	389.0 (0)	2.9 (0)	0 (0)

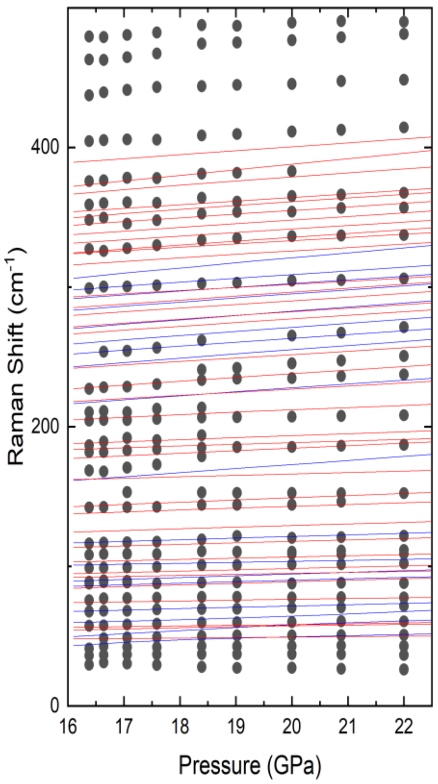

Figure S5. Pressure dependence of the experimental (symbols) and theoretical (lines) Raman frequencies of $Pb_6Ga_{10}S_{21}$ during compression. The red (blue) lines correspond to the theoretical A_g (B_g) modes.

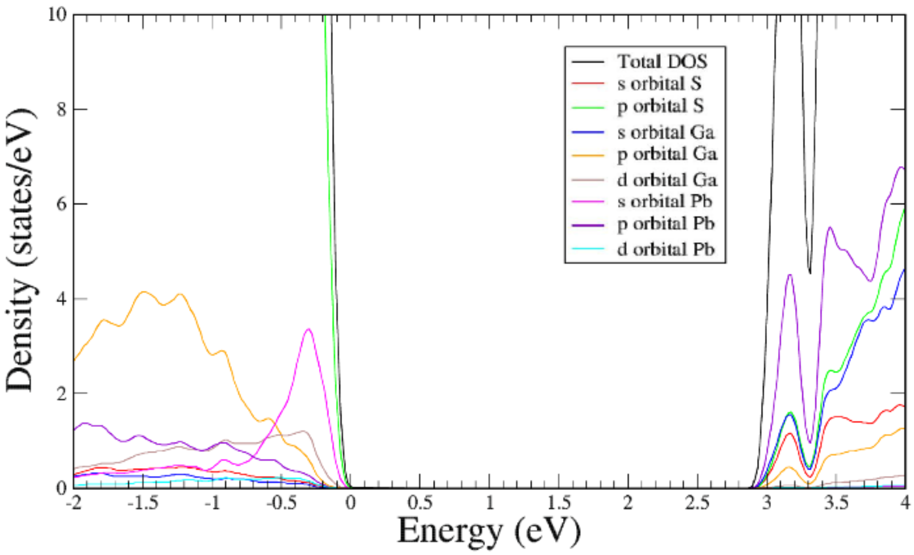

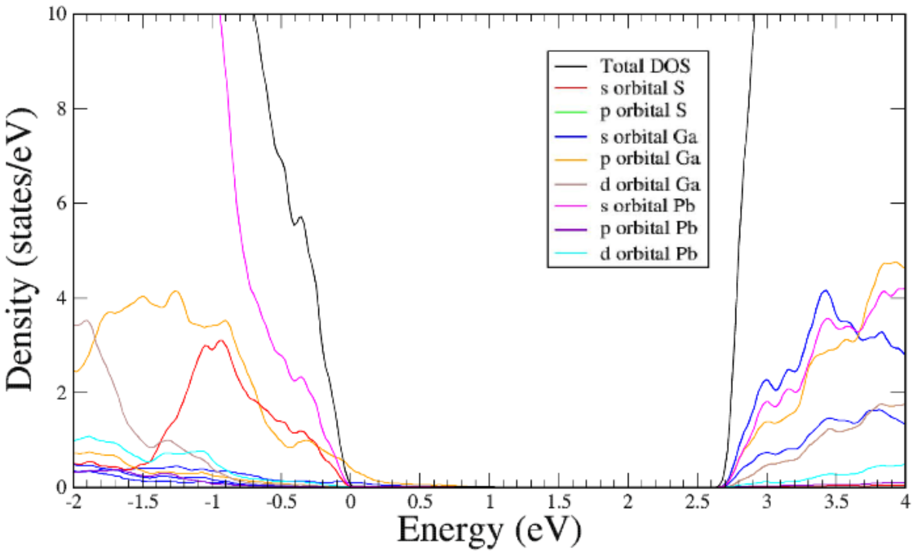

Figure S6. (Top) Zoom of the total and partial electronic density of states of $PbGa_2S_4$ at 0 GPa. (Bottom) Zoom of the total and partial electronic density of states of $Pb_6Ga_{10}S_{21}$ at 15.3 GPa

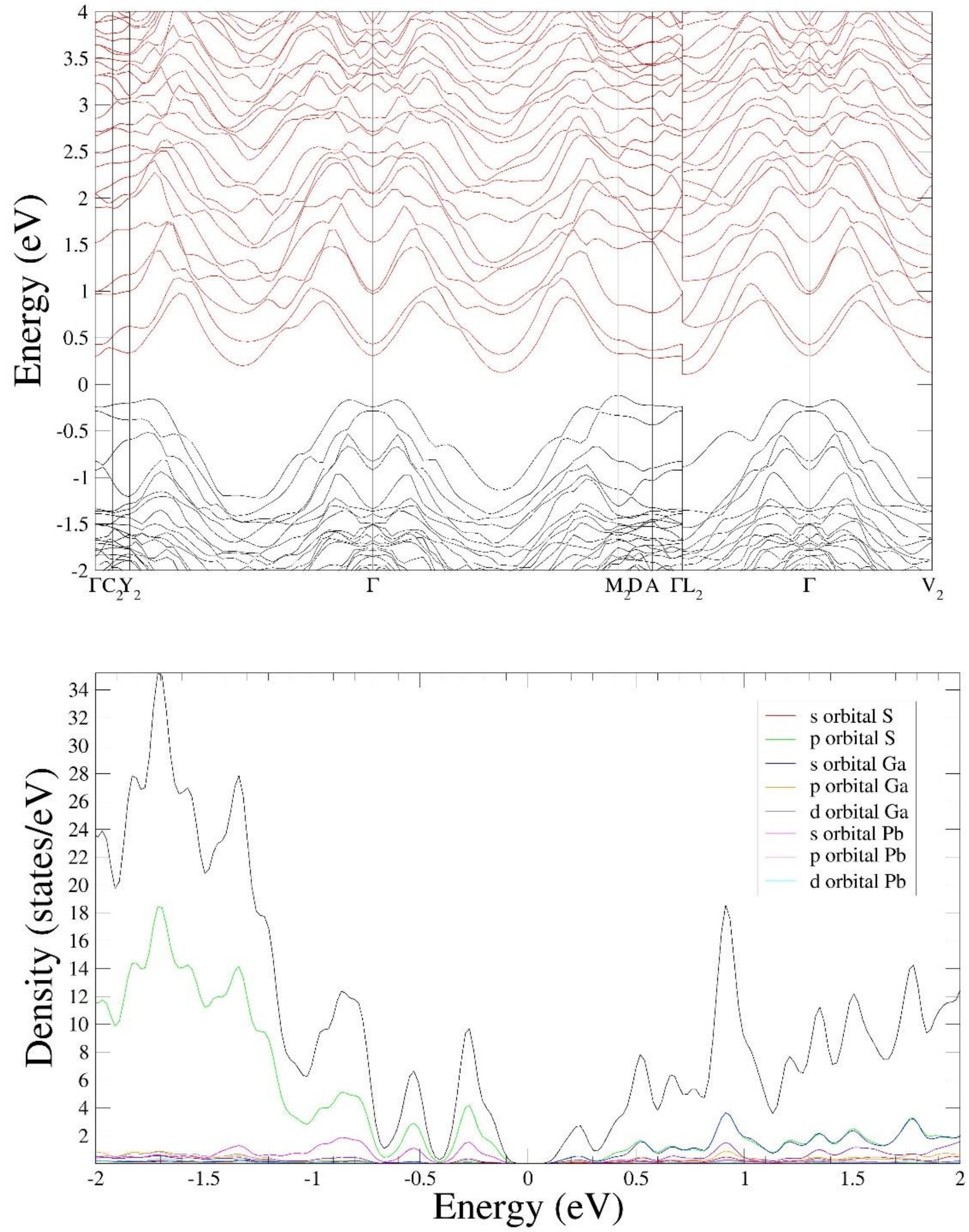

Figure S7. (Top) Electronic band structure of $Pb_6Ga_{10}S_{21}$ at 15.3 GPa. (Bottom) Total and partial electronic density of states at 15.3 GPa

Bibliography

[S1] CrysAlisPro Software System, Rigaku Oxford Diffraction, (2020).

[S2] Sheldrick, G.M., Crystal structure refinement with ShelXL, *Acta Cryst.*, (2015), **C71**, 3-8.

[S3] Sheldrick, G.M., ShelXT-Integrated space-group and crystal-structure determination, *Acta Cryst.*, (2015), **A71**, 3-8.

[S4] O.V. Dolomanov and L.J. Bourhis and R.J. Gildea and J.A.K. Howard and H. Puschmann, Olex2: A complete structure solution, refinement and analysis program, *J. Appl. Cryst.*, (2009), **42**, 339-341.
